# Genetic Diversity of Blueberry Genotypes Estimated by Antioxidant Properties and Molecular Markers

**DOI:** 10.3390/antiox10030458

**Published:** 2021-03-15

**Authors:** Dhrumit S. Bhatt, Samir C. Debnath

**Affiliations:** 1 Department of Biology, Memorial University of Newfoundland, 232 Elizabeth Avenue, St. John’s, NL A1B 3X9, Canada; dhrumit.bhatt@gmail.com; 2 St. John’s Research and Development Centre, Science and Technology Branch, Agriculture and Agri-Food Canada, Bldg. 25, 204 Brookfield Road, St. John’s, NL A1E 6J5, Canada

**Keywords:** antioxidant activity, biochemical and molecular diversity, blueberries, genetic structure, phenolics, flavonoids, molecular markers

## Abstract

Blueberries (*Vaccinium* spp.) have gained much attention worldwide because of their potential health benefits and economic importance. Genetic diversity was estimated in blueberry hybrids, wild clones and cultivars by their antioxidant efficacy, total phenolic and flavonoid contents, and express sequence tag–simple sequence repeat (SSR) (EST–SSR), genomic (G)–SSR and express sequence tag–polymerase chain reaction (EST–PCR) markers. Wide diversity existed among the genotypes for antioxidant properties, with the highest variation for DPPH radical scavenging activity (20-fold), followed by the contents of total flavonoids (16-fold) and phenolics (3.8-fold). Although a group of 11 hybrids generated the maximum diversity for antioxidant activity (15-fold), wild clones collected from Quebec, Canada, had the maximum variation for total phenolic (2.8-fold) and flavonoid contents (6.9-fold). Extensive genetic diversity was evident from Shannon’s index (0.34 for EST–SSRs, 0.29 for G–SSR, 0.26 for EST–PCR) and expected heterozygosity (0.23 for EST–SSR, 0.19 for G–SSR, 0.16 for EST–PCR). STRUCTURE analysis separated the genotypes into three groups, which were in agreement with principal coordinate and neighbour-joining analyses. Molecular variance suggested 19% variation among groups and 81% among genotypes within the groups. Clustering based on biochemical data and molecular analysis did not coincide, indicating a random distribution of loci in the blueberry genome, conferring antioxidant properties. However, the stepwise multiple regression analysis (SMRA) revealed that 17 EST–SSR, G–SSR and EST–PCR markers were associated with antioxidant properties. The study is valuable to breeding and germplasm conservation programs.

## 1. Introduction

Blueberry is an economically and medicinally high-value crop that belongs to the genus *Vaccinium* L., which contains about 400–500 species native to countries all over the world except Antarctica and Australia [1,2]. The five major groups of blueberries grown commercially include (1) lowbush (*V. angustifolium* Ait.; 2n = 4x = 48), (2) highbush (*V. corymbosum* L.; 2n = 4x = 48), (3) half-high (the product of hybridization between highbush and lowbush blueberries), (4) southern highbush (hybrids between *V. corymbosum* and mostly *V. darrowi* Camp and/or *V. ashei* Reade) and (5) Rabbiteye (*V. virgatum* Ait.; syn. *V. ashei*; 2n = 6x = 72) [3]. Blueberries are consumed fresh or in other commercially processed forms mainly for their high antioxidant activity, which fight off harmful radicals in the body. The high antioxidation activity is due to high concentrations of anthocyanins, flavonoids and phenolic acids. These phenolic compounds are linked to an improvement of night vision, prevention of macular degeneration, anticancerous activity and reduction in heart disease [4,5]. The clinical benefits of blueberry are not only limited to its fruits; blueberry leaves have also been shown to possess antidiabetic [6] and antimicrobial activities [7] and have been used as a traditional remedy for the treatment of diabetic symptoms [8,9].

Because of their health benefits, there has been a steep surge in demand for high-quality blueberries. Therefore, efforts were made at St. John’s Research and Development Centre of Agriculture and Agri-Food Canada (AAFC) in Newfoundland and Labrador, Canada, in 1999 to create cultivars that can sustain in cool climates [10]. Earlier efforts to improve blueberry involved breeding and selection, where suitable and novel traits, such as fruit quality, tolerance to drought and cold and disease resistance, were pooled together by interspecific hybridization [11]. The knowledge of genetic diversity in available berry germplasm can facilitate reliable and fast identification of genotypes. This knowledge can be invaluable for conservation efforts, designing specific breeding programs, selecting various combinations of parents with high diversity to create offspring with maximum genetic variation and introducing desirable genes into the available germplasm for berry improvement [3].

It has been a regular practice to use molecular markers for diversity analyses in blueberries, including restriction fragment length polymorphism (RFLP) [12], random amplified polymorphic DNA (RAPD) [13,14], amplified fragment length polymorphism (AFLP) [13], inter simple sequence repeat (ISSR) [15,16], simple sequence repeat (SSR) [10,17,18,19], express sequence tag–polymerase chain reaction (EST–PCR) and cleaved amplified polymorphic sequences (CAPSs) derived from EST–PCR markers [10,19]. EST–PCR markers were initially used in highbush blueberries [20] and have also been found appropriate for genetic association studies on lowbush [10,21,22] and rabbiteye blueberries [23]. While SSRs are repeats of short nucleotide sequences, mostly with six or less bases, EST–PCRs are sequence-tagged site (STS) markers that use precise primers of ~20-mer primers in expressed sequence tags (ESTs) [23].

The objective of the present study is to examine the antioxidant properties, genetic diversity and relationship between antioxidant properties and molecular diversity in two groups of selected hybrids developed under an on-going blueberry genetic enhancement program, four groups of lowbush blueberry wild clones and a group of blueberry cultivars. Total antioxidant activity (TAA), and total phenolic (TPC) and flavonoid contents (TFC) were estimated to check biochemical diversity. Three types of DNA-based molecular markers, including EST–SSR, genomic (G)–SSR and EST–PCR, were used for a detailed molecular biodiversity study that will improve the efficiency in cultivar development, variety protection and registration, along with phenotypic selection. Comparative efficiency of molecular markers with those of biochemical diversity has not been reported in blueberries.

## 2. Materials and Methods

### 2.1. Plant Materials

A total of 28 blueberry hybrids (Table 1), 36 lowbush blueberry wild clones (Table 2), and six lowbush, half-high, and highbush blueberry cultivars (Table 1) were used in the study. While the hybrids, designated as HB1 to HB28, were selected from crosses between lowbush and half-high/highbush blueberries, the wild clones, named BC1 to BC36, were from four Canadian provinces: Newfoundland and Labrador (NL), Prince Edward Island (PE), Quebec (QC) and New Brunswick (NB). Each clone implied one wild plant selected based on vigour, berry colour, berry size and yield per plant and apparently free from diseases and insects. The distance between any two plants in the same place was above 10 m. Each selected plant was phenotypically different from the other and was considered an individual clone [10].

The lowbush blueberry cultivar Fundy (FUN), a selection from open-pollinated seedlings of cultivar Augusta, was developed at Kentville Research and Development Centre, AAFC, NS, Canada [24]. The parentages of highbush blueberry cultivar Polaris (POL) and four half-high blueberry cultivars, Patriot (PAT), Chippewa (CHIP), St. Cloud (STC) and Northblue (NOB), are presented in Table 1. The lowbush blueberries were less than 0.5 m in height, and the highbush blueberries were 2–2.5 m tall. Half-high and hybrid blueberries used in the study were of intermediate height, between lowbush and highbush blueberries. All genotypes were grown and maintained in a greenhouse in 6 L plastic pots containing a 2:1 peat:perlite mixture, under natural light conditions (maximum PPF 90 µmol m^−2^ s^−1^) at 20 ± 2 °C and 85% relative humidity. Normal cultural practices were followed to maintain the plants [10]. In a replication of three from each plant, fresh young leaves were collected, shock-frozen with liquid nitrogen, and stored at −80 °C until DNA and phenolic extraction for molecular and biochemical analyses, respectively.

### 2.2. Preparation of Leaf Extracts

Briefly, 500 mg of shock-frozen leaves of each genotype was homogenized in a FastPrep-24 Tissue and Cell Homogenizer (MP Biomedicals, Irvine, CA, USA) containing a solution of 80% aqueous acetone and 0.2% formic acid (1:4 g/mL) [28,29]. The homogenate was retained at 4 °C, with slow agitation for 30 min, followed by centrifugation at 20,000× *g* at 4 °C for 15 min using an Allegra 64R (Beckman Coulter Inc., Palo Alto, CA, USA), and the supernatant was collected. Extraction was performed twice more with the pallets, and the supernatant was mixed with the original crude extract. The extracts were saved in an ultralow freezer (Thermo Scientific, Burlington, ON, Canada) for further determination of antioxidant capacity and total phenolic and flavonoid contents. All chemical analyses were conducted thrice with each sample, and mean values were used for analysis.

### 2.3. Total Antioxidant Activity (TAA)

Free radical scavenging activity was estimated as percentage inhibition of radicals. 2,2-Diphenyl-1-picrylhydrazyl (DPPH) is an artificially stabilized free radical [29]. An aliquot of diluted extract or gallic acid standard solution (5 mg/mL; ≥98% purity) was added to 1.7 mL DPPH methanolic solution (0.06 mM), mixed thoroughly, and kept in the dark for 20 min at room temperature. The mixture’s absorbance was examined at 517 nm using an Ultrospec 4300 Pro UV–visible spectrophotometer (Amersham Biosciences Corp. San Francisco, CA, USA). Blank was prepared using aqueous acetone (80%) mixed with the DPPH solution. The gallic acid standard curve (at ≥98% purity) was prepared, and the linearity of the gallic acid standard curve (r^2^ = 0.98) was obtained in the range of 20–80 μg/mL. The results were expressed as milligrams of gallic acid equivalents (GAE) per gram of fresh leaf (mg GAE/g fl). The following formula was used to calculate percentage inhibition [30]:

% *Radical scavenging activity* = [(*Absorbance _(Blank)_* − *Absorbance _(Extract)_*)/*Absorbance _(Blank)_*] × 100

### 2.4. Total Phenolic Content (TPC)

An optimized Folin–Ciocalteu method [28,31] was used to estimate total phenolic content. Folin–Ciocalteu reagent (100 μL) was added to the diluted leaf extract (100 μL; 200 μg/mL), and 200 μL of 20% saturated (*w/v*) sodium carbonate was added to it after 5 min, followed by 1.5 mL distilled water. The mixture was incubated in the dark for 35 min at room temperature and centrifuged at 4000× *g* for 10 min in Allegra 64R. The absorbance of the gallic acid standard solution (5 mg/mL) and test samples were measured with Ultrospec 4300 Pro at 725 nm wavelength after 3 min. The absorbance values were recorded linearly of the standard calibration curve (r^2^ = 0.98) for the gallic acid standard solution (5 mg/mL; ≥98% purity), taken in a range of 2.5–10 μg/mL, and outcomes are presented as mg GAE/g fl.

### 2.5. Total Flavonoid Content (TFC)

Total flavonoid content was estimated using colorimetric assay [32] following Goyali et al. [28]; 500 µL of sample extract was added to 2 mL distilled water, followed by 150 mL of 5% (*w/v*) sodium nitrate, to which 150 mL of 10% (*w/v*) aluminum chloride was added after 5 min. Then, 1 M sodium hydroxide solution (1 mL) was added to the mixture after 6 min of incubation at room temperature. The mixture was diluted with 1.2 mL distilled water, and the absorbance was measured at 510 nm using Ultrospec 4300 Pro. Catechin solution (1 mg/mL) was used in a range of 20–200 μg/mL for standard curve calibration (r^2^ = 0.99), and the total flavonoid content was calculated as milligrams of catechin equivalents per gram of fresh leaves (mg CE/g fl).

### 2.6. DNA Extraction, PCR Amplification, and Electrophoresis

Briefly, 200 mg of stored young leaf sample was used to isolate DNA using a DNeasy Plant Mini Kit (Qiagen GmbH, Hilden, Germany), with some modifications [10]. Frozen leaves were homogenized with 550 µL buffer AP1 with the help of FastPrep-24 Tissue and Cell Homogenizer (MP Biomedicals, Santa Ana, CA, USA). Then, 4 µL of RNase A was added to the homogenized mixture and incubated for 60 min at 65 °C, and contents were mixed several times at regular intervals. Further steps were followed, as per manufacturer instructions. The purity and concentration of DNA were measured spectrophotometrically (Ultrospec 2000, Pharmacia Biotech, Cambridge, UK). DNA with an A260/A280 absorbance ratio of 1.7–2.1 was diluted (concentration: 10 ng mL^–1^) and used as template DNA for PCR reactions [10]. A total of 10 EST–SSR (CA23, CA112, CA169, CA236, CA421, CA483, CA787, NA800, NA961 and NA1040), eight genomic SSR (G–SSR; VCC_B3, VCC_I2, VCC_I8, VCC_J1, VCC_J3, VCC_J9, VCC_K4 and VCC_S10) and eight EST–PCR primer pairs (CA21, CA54, CA227, CA287, CA791, CA1029, CA1423 and NA27; Table S1) that were found effective for blueberries [19,21] were obtained from Integrated DNA Technologies, Inc. (IDT), Coralville, Iowa, USA. The EST–PCR primer pairs were developed from floral buds of cold-acclimated (CA) and nonacclimated (NA) highbush blueberries [20]. SSR markers were the derivatives of SSR-enriched genomic libraries and EST libraries were constructed from highbush blueberries [19]. The annealing temperature of all primers was standardized using temperature gradient PCR that ranged from 49 °C for the EST-SSR primer pair CA112 to 62 °C for EST-SSR primer pair NA800 and G-SSR primer pairs VCC_B3, VCC_B12, VCC_J9 and VCC_K4 (Table S1).

The PCR was carried out in an optimized amplification reaction mixture (25 µL) containing 20 ng of template DNA, 1× PCR buffer (1.5 mM MgCl2, pH 8.7; Qiagen), 200 µM of each deoxynucleotide triphosphate (dNTP), 0.2 µM of each of the 20 forward and reverse primers and 0.63 unit of Taq DNA polymerase (Qiagen). Mastercycler ep Gradient S (Eppendorf AG, 22331 Hamburg, Germany) was used to amplify DNA, which was programmed for a 10-min initial “hot start” denaturation step at 94 °C, and then 40 cycles of 40 s of a denaturation step at 92 °C, a 70-s annealing step at an appropriate annealing temperature and a 2-min extension step at 72 °C. The final extension step was at 72 °C for 10 min, and then the sample was held at 4 °C. The amplified DNA products were separated by electrophoresis using 2% agarose 3:1 high-resolution blend (HRB; Ameresco, Solon, OH) gel, precasted with 2× tris-borate ethylene diamine tetraacetic acid buffer and 1× GelRed nucleic acid stain (Biotium Inc., Hayward, CA 94545, USA) solution, along with a low range 100 base pair (bp) DNA ladder and a midrange 1 kb DNA ladder (Norgen Bioteck Corp., Thorold, ON, Canada). UV light enabled visualization, scoring, and recording banding patterns in a transilluminating gel documentation system (InGenius 3, Syngene, Beacon House, Cambridge, UK). The length of the DNA fragment was calculated by Gene Tools software (Syngene) by comparison with standard size marker mobility [22].

### 2.7. Data Collection and Statistical Analysis

Data for antioxidant activity, phenolic, and flavonoid contents are presented as mean value ± standard deviation (SD) of three replications. The TAA result among groups was statistically evaluated by variance analysis (ANOVA), and Tukey’s test was employed for comparing treatment means at a critical difference (*p*) of ≤0.05. Because the residuals of TPC and TFC among groups followed non-normal distribution, a violation of one of the preconditions of ANOVA, the observations were statistically evaluated by a nonparametric Kruskal–Wallis test [33], with the significance value fixed at ≤0.05. Similarly, the residuals of TAA, TPC, and TFC among the individuals followed non-normal distribution; the observations were statistically evaluated by a nonparametric Kruskal–Wallis test, with the significance value fixed at ≤0.05. The correlation coefficient (r), coefficient of determination (r^2^), and linear regression between TPC and TFC, TPC and TAA, and TFC and TAA were analyzed at a confidence interval of 95%. To eliminate the effects of different scales of measurement, the biochemical data were standardized by subtracting mean values from the original values, followed by division with SD [34]. A Euclidean dissimilarity distance matrix was generated using these standardized values. The agglomerative hierarchical clustering (AHC) method, an algorithm that works on the dissimilarities between various individuals and groups them, was used to generate an unweighted pair group average (UPGMA) dendrogram based on the Euclidean dissimilarity distance matrix of antioxidant activity and phenolic and flavonoid content data. Principal component analysis (PCA), a multivariate technique that analyzes data matrices of several correlated quantitative dependent variables, was performed for individuals, based on biochemical data, as well as for biochemical components [35]. The above analysis was performed using XLSTAT 2020 version 22.3.21.0 (©Addinsoft, New York, NY 10001, USA).

EST–SSR, G–SSR, and EST–PCR markers, which can discriminate between homozygous and heterozygous individuals, are codominant in nature. In polyploid plants like blueberry, it is very difficult to perform codominant scoring of alleles in heterozygote samples as it is extremely difficult to calculate the number of alleles present at a particular locus from band intensities; the only suggested way of scoring is to record the absence or presence of an allele denoted as 0 or 1, respectively, in a matrix, and all bands from one primer are treated as alleles and one locus [36,37].

Indices, including polymorphic information content (*PIC*) [38], effective multiplex ratio (EMR) [39], discrimination power (*D*) [40] and resolving power (*R*) [41], were calculated using the program Online Marker Efficiency Calculator (iMEC) [42]. These indices give an idea about the primer’s ability to discriminate among genotypes and the primer system’s overall utility. The *PIC* is a value that reflects the marker’s ability to detect polymorphism within the population. *PIC* gives an approximation of a primer’s discrimination capacity based on the number of alleles that are expressed and the respective allelic frequencies. This was used to evaluate the level of informativeness of each primer (high, *PIC* > 0.5; moderate, 0.5 > *PIC* > 0.25; low, *PIC* < 0.25) [38]. The associated value EMR was calculated as the product of a total number of polymorphic loci and the portion of polymorphic loci over a total number of loci [39]:

EMR = n_p_.n_p_/n

where n_p_ is the number of polymorphic loci, and n is the total number of loci. Therefore, the higher the value of EMR, the more competent the primer is. 

MI is another associated statistical parameter used to evaluate a marker system [39]. It was calculated as follows:

MI = PIC.EMR

Primer discrimination power, *D*, is defined as the probability that two randomly chosen individuals have different banding patterns and are, therefore, differentiable [40]. This was calculated as follows:

D = 1 − C

where C is the confusion probability. For the i-th pattern of the given j-th primer, present at frequency pi in a set of varieties, C = Σ ci = Σ pi Npi-1/N−1, where for N individuals, C is equal to the sum of all ci for all of the patterns generated by the primer. 

Resolving power, *R*, was calculated as follows:

R = ∑ Ib

where Ib or band informativeness is denoted on a scale of 0 or 1 and is described as Ib = 1 − (2 × |0.5 − p|); p is defined as the samples’ portion of the observed band. The resolving power or the primer’s capability to differentiate between genotypes can be represented by the sum of these adjusted values for all generated bands [41].

To compare diversity among blueberry genotypes, indices such as percentage of polymorphic loci (*PL*), the observed (*Na*) and effective number of alleles (*Ne*) [43], expected heterozygosity/Nei gene diversity index (*He*) [44] and Shannon’s information index of diversity (*I*) [45] were calculated using GenAlEx version 6.5 [46].

A Bayesian clustering approach for population structure analysis was used for 70 genotypes using the STRUCTURE program ver. 2.3.4 (https://web.stanford.edu/group/pritchardlab/structure_software/release_versions/v2.3.4/html/structure.html (accessed in September 2020)). The software uses a Markov Chain Monte Carlo (MCMC) estimation to determine the number of subpopulations (ΔK) [47]. Using this model, some populations (K) were presumed to be present, and each of them was characterized by a set of allele frequencies at every locus. Genotypes in the sample were assigned to clusters (populations) or jointly to more populations if their genotypes indicated that they were admixed. Every locus was thought to be independent, and each K population was presumed to follow the Hardy–Weinberg equilibrium. The ΔK method [48] was used to determine the most likely number of K. The number of genetically different clusters (K) was kept to range 1 to 10, with five independent runs, followed by a burn-in length of 100,000 and 100,000 iterations. POPHELPER, an R package, and a web server (http://royfrancis.github.io/pophelper/, accessed on 1 September 2020) were used to estimate the number of population clusters and their visualization [49].

The software DARwin 6.0.9 [50] was used to depict phylogenetic trees with EST–SSR, G–SSR, and EST–PCR markers using unweighted neighbour-joining (NJ). Jaccard’s coefficient [51] was used to calculate the dissimilarity matrix, with 30,000 bootstraps. GenAlEx version 6.5 [46] was used for principal coordinate analysis (PCoA) and hierarchical analysis of molecular variance (AMOVA). For AMOVA, the blueberry genotypes were divided into seven groups, out of which four groups were comprised of wild clones collected from four Canadian provinces (NL, PE, QC, and NB). Group 5 consisted of six blueberry cultivars, Group 6 of 11 hybrids from the first cross (HB1–11), and Group 7 of 17 hybrids from the second cross (HB12–28).

A mantel test [52,53] was performed to check the correlation between the Euclidean dissimilarity distance matrix generated using standardized values of biochemical data in XLSTAT 2020 Version: 22.3.21.0 (©Addinsoft, New York, NY 10001, USA) and genetic distance matrices of EST–SSR, G–SSR, EST–PCR, and all primers combined, generated using GenAlEx version 6.5 [46].

The association between EST–SSR, G–SSR and EST–PCR markers and biochemical attributes of blueberry leaf extracts (TAA, TPC and TFC) was estimated by stepwise multiple regression analysis (SMRA) [54] using SPSS version 27 (IBM Corp., Armonk, NY 10504–1722, USA). The biochemical components were treated as dependent variables, and molecular markers were treated as independent variables. The F-value criteria was set between 0.045–0.099 for the inclusion or removal of independent variables for regression [55].

## 3. Results

### 3.1. Biochemical Diversity 

#### 3.1.1. Total Antioxidant Activity (TAA)

Total antioxidant activity was highly diverse (*p* < 0.015) in the present material (Table 3 and Table S2). The values varied 20-fold among all genotypes, with BC6 and BC22 having the highest TAA (5.82 ± 0.03 mg GAE/g fl), followed by BC13 (5.45 ± 0.09 mg GAE/g fl), while HB6 had the lowest (0.29 ± 0.10 mg GAE/g fl). The variation for TAA was the highest among genotypes in Cross 1 (15 times) and lowest in Cross 2 (2.04 times), followed by cultivars (2.09 times), among all groups. The NB wild clones had the highest average TAA value (4.29 ± 1.06), followed by CVs (4.23 ± 1.10 mg GAE/g fl) and Cross 2 hybrids (4.08 ± 0.89 mg GAE/g fl). Hybrids in Cross 1 had the lowest average TAA value (2.55 ± 1.28 mg GAE/g fl) among all the groups. In NL clones, the highest TAA value was observed in genotype BC6 (5.82 ± 0.03 mg GAE/g fl) and the lowest in BC9 (2.45 ± 0.07 mg GAE/g fl). The TAA values for PE clones ranged from 1.14 ± 0.08 for BC12 to 5.45 ± 0.09 for BC13. In wild QC clones, the TAA was highest in BC22 (5.82 ± 0.03 mg GAE/g fl) and lowest in BC28 (1.14 ± 0.05 mg GAE/g fl). The value of TAA among NB wild clones ranged from 1.84 ± 0.05 mg GAE/g fl for BC29 to 5.13 ± 0.07 mg GAE/g fl for BC34. The values of TAA among NB wild clones ranged from 1.84 ± 0.05 mg GAE/g fl for BC29 to 5.13 ± 0.07 mg GAE/g fl for BC34. The average TAA value for eight NB wild clones was the highest (4.29 ± 1.06 mg GAE/g fl) among all groups (Table 3). For cultivars, the highest TAA value was observed in highbush cultivar POL (5.19 ± 0.03 mg GAE/g fl), followed by FUN (5.12 ± 0.07 mg GAE/g fl), and the lowest in half-high cultivar STC (2.48 ± 0.08 mg GAE/g fl). The value for TAA in Cross 1 ranged from 0.29 ± 0.10 for HB6 to 4.48 ± 0.04 for HB11 and, in Cross 2, it ranged from 2.52 ± 0.07 mg GAE/g fl for HB16 to 5.13 ± 0.05 mg GAE/g fl for HB21.Two NL (BC6: 5.82 ± 0.03 mg GAE/g fl; BC13: 5.45 ± 0.09 mg GAE/g fl) and one QC (BC22: 5.82 ± 0.03 mg GAE/g fl) clones possessed more TAA than all cultivars (Table S2). 

The value of TAA among NB wild clones ranged from 1.84 ± 0.05 for BC29 to 5.13 ± 0.07 for BC34. The average TAA value for eight NB wild clones was the highest (4.29 ± 1.06) among all groups (Table 3). For cultivars, the highest TAA value was observed in highbush cultivar POL (5.19 ± 0.03) and the lowest in half-high cultivar STC (2.48 ± 0.08). The value for TAA in Cross 1 ranged from 0.29 ± 0.10 for HB6 to 4.48 ± 0.04 for HB11, and, in Cross 2, it ranged from 2.52 ± 0.07 for HB16 to 5.13 ± 0.05 for HB21.

#### 3.1.2. Total Phenolic Content (TPC)

TPC estimations using Folin–Ciocalteu reagent were highly diverse (*p* < 0.0003). The variation was 3.83 times among all genotypes, with a range from 0.06 ± 0.01 mg GAE/g fl for BC21 to 0.24 ± 0.01 mg GAE/g fl for BC2 (Table 3 and Table S2). The 10 clones collected from NL had the highest average TPC value (0.16 ± 0.05), followed by the CV group (0.13 ± 0.03) and PE wild clones (0.12 ± 0.02), among all groups. In NL clones, the TPC values ranged from 0.09 ± 0.01 mg GAE/g fl for BC8 to 0.24 ± 0.01 mg GAE/g fl for BC2. Among PE clones, the lowest TPC was observed in BC20 (0.09 ± 0.01 mg GAE/g fl) and the highest in BC11 and BC18 (0.15 ± 0.01 mg GAE/g fl and 0.15 ± 0.00 mg GAE/g fl, respectively). TPC for eight QC wild clones varied from 0.06 ± 0.01 mg GAE/g fl (BC21) to 0.18 ± 0.01 mg GAE/g fl (BC22). In NB wild clones, the highest TPC was observed for BC36 (0.15 ± 0.01 mg GAE/g fl) and lowest for BC29 (0.08 ± 0.00 mg GAE/g fl). The value for TPC among cultivars was the highest for highbush cultivar POL (0.16 ± 0.01 mg GAE/g fl) and the lowest for half-high cultivar STC (0.09 ± 0.00 mg GAE/g fl). The TPC value for Cross 1 and Cross 2 ranged from 0.07 ± 0.01 mg GAE/g fl (HB3) to 0.16 ± 0.01 mg GAE/g fl (HB1, 11) and from 0.08 ± 0.00 mg GAE/g fl (HB19) to 0.12 ± 0.00 mg GAE/g fl (HB14), respectively. Among all genotypes, four NL clones (BC2, 0.24 ± 0.01; BC3, 0.20 ± 0.02, BC5, 0.20 ± 0.02; BC6, 0.20 ± 0.01) and one QC clone (BC22, 0.18 ± 0.01) possessed higher TPC than the cultivars. 

#### 3.1.3. Total Flavonoid Content (TFC)

The results for TFC are displayed in Table 3 and Table S2. The wild clones, cultivars and hybrids showed wide variation (15.63 times) among themselves for TFC (*p* < 0.0003), ranging from 0.64 ± 0.02 CE/g fl to 9.99 ± 0.11 CE/g fl for the cultivar NOB and NL wild clone BC2, respectively. The wild clones from NL had the highest average TFC (5.26 ± 2.49 CE/g fl), followed by PE (2.14 ± 0.79 CE/g fl) and QC (1.88 ± 1.59 CE/g fl) wild clones, among all groups. The average values of TFC were the lowest in Cross 2 (1.41 ± 0.29 CE/g fl), and it was followed by Cross 1 (1.39 ± 0.18 CE/g fl). For NL clones, the TFC value was the highest in BC2 (9.99 ± 0.11 CE/g fl) and the lowest in BC8 (2.00 ± 0.01 CE/g fl). The values of TFC for PE clones ranged from 3.77 ± 0.09 CE/g fl for BC18 to 1.22 ± 0.02 CE/g fl for BC15. The highest and lowest values of TFC for QC clones were found in BC22 (5.56 ± 0.04 CE/g fl) and BC21 (0.81 ± 0.02 CE/g fl). TFC for eight NB wild clones ranged from 1.19 ± 0.02 CE/g fl (BC29) to 2.70 ± 0.05 CE/g fl (BC34). The value for TFC among cultivars was the highest for lowbush cultivar FUN (2.83 ± 0.02) and the lowest for half-high cultivar NOB (0.64 ± 0.02 CE/g fl). The TFC value for Cross 1 and Cross 2 ranged from 1.04 ± 0.03 CE/g fl (HB1) to 1.71 ± 0.02 CE/g fl (HB3) and from 0.87 ± 0.04 CE/g fl (HB19) to 1.95 ± 0.03 CE/g fl (HB12), respectively. While eight NL clones (BC1–7, 10) were found superior, with higher TPC, ranging from 3.49 ± 0.03 mg CE/g fl to 9.99 ± 0.11 mg CE/g fl, than those of the cultivars, there were two clones—one PE clone (BC18, 3.77 ± 0.09 mg CE/g fl) and one QC clone (BC22, 5.56 ± 0.04)—that had higher TFC than the cultivars (S2).

#### 3.1.4. Relationship Among Antioxidant Properties

**Figure 1:**
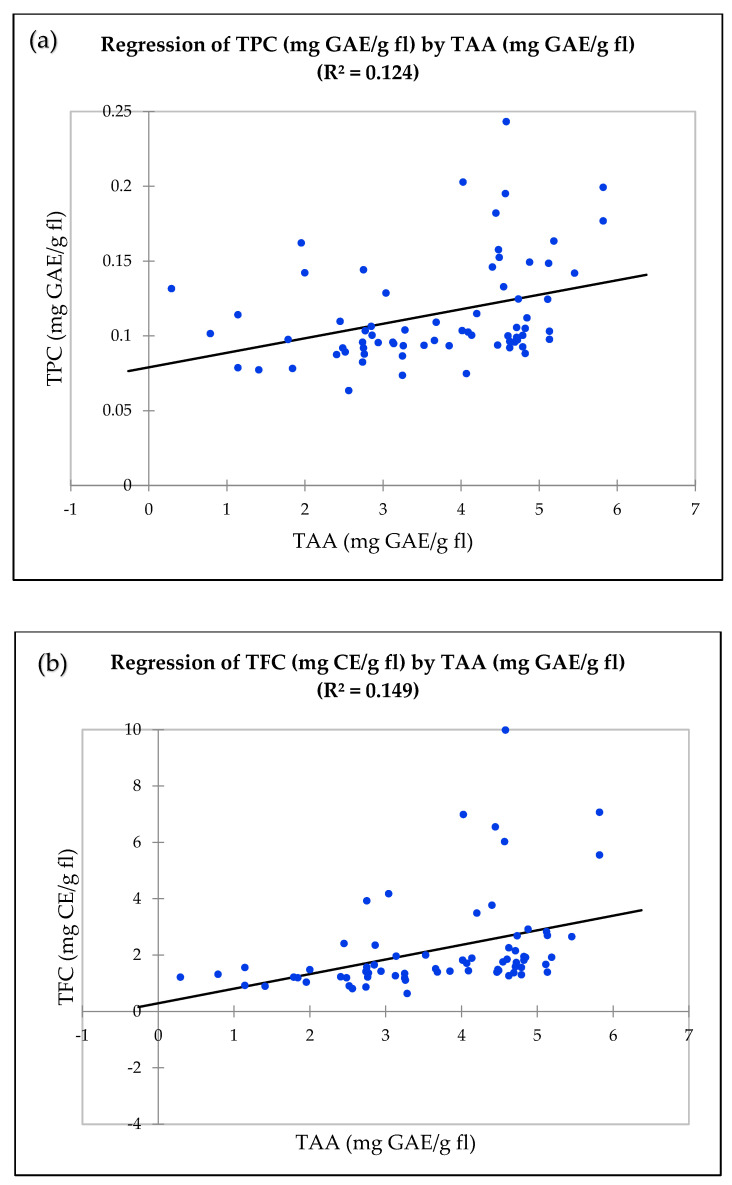

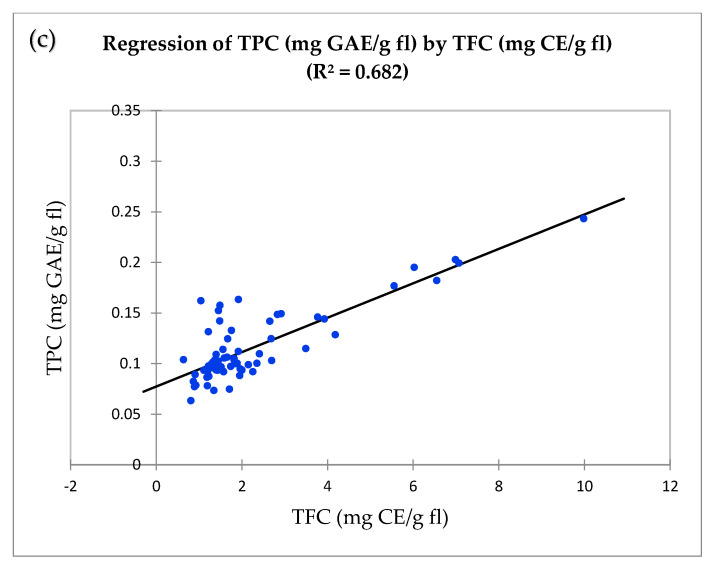
Linear regression between antioxidant properties in wild clones, cultivars, and hybrids. (**a**) Total antioxidant activity by DPPH (mg GAE/g fl) and total phenolic content (mg GAE/g fl). (**b**) Total antioxidant activity by DPPH (mg GAE/g fl) and total flavonoid content (mg CE/g fl). (**c**) Total phenolic content (mg GAE/g fl) and total flavonoid content (mg CE/g fl).

To evaluate the relationships between TAA, TPC and TFC, linear regression was performed. There was a significant relationship observed between TAA and TPC (r^2^ = 0.124, Figure 1a), TAA and TFC (r^2^ = 0.149, Figure 1b), and TFC and TPC (r^2^ = 0.682, Figure 1c). The Pearson correlation coefficient between TFC and TPC (r = 0.826) was significantly higher, followed by TAA and TFC (r = 0.387) and TAA and TPC (r = 0.352) (Table S3).

#### 3.1.5. Cluster Analysis of Antioxidant Properties

##### Agglomerative Hierarchical Clustering (AHC)

**Figure 2:**
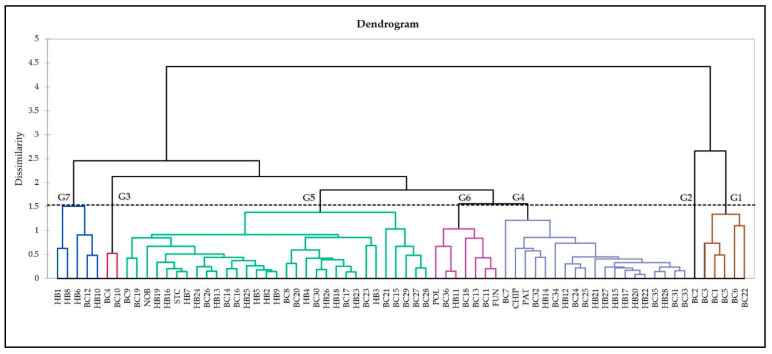
AHC dendrogram by the unweighted pair-group average (UPGMA) method, based on a Euclidean dissimilarity distance matrix of antioxidant activity, phenolic and flavonoid content data for 70 genotypes.

An unweighted pair group average (UPGMA) dendrogram was generated based on the Euclidean dissimilarity distance matrix of antioxidant activity, phenolic, and flavonoid content data. The dendrogram (Figure 2) shows the presence of two clades (I and II), subdivided into seven major groups (Clade I: G1, 2; Clade II: G3–7) that are neither grouped according to genotype nor geographic distribution. Group 1 consists of four NL (BC1, 3, 5, 6) and one QC (BC22) wild clones. Group 2 only has one NL wild clone (BC2). NL wild clones BC4 and BC10 make Group 3. Group 4 is comprised of 19 genotypes, including one NL (BC7), two QC (BC24, 25), and five NB (BC31–35) wild clones, two half-high cultivars (PAT, CHIP), and nine individuals from Cross 2 (HB12, 14, 15, 17, 20–22, 27, 28). The largest group, Group 5, consists two NL (BC8, 9), six PE (BC14–17, 19, 20), five QC (BC21, 23, 26–28), and two NB (BC 29, 30) wild clones, two half-high cultivars (STC, NOB), 14 hybrids, with six belonging to Cross 1 (HB2–5, 7, 9) and eight to Cross 2 (HB13, 16, 18, 19, 23, 24–26). Group 6 is comprised of three PE (BC11, 13, 18) and one each from NB (BC36), lowbush cultivar (FUN), and Cross 1 (HB11) individuals. Group 7 predominately consists of individuals from Cross 1 (HB1, 6, 8, 10) and one PE wild clone (BC12).

###### Principal Component Analysis (PCA)

**Figure 3:**
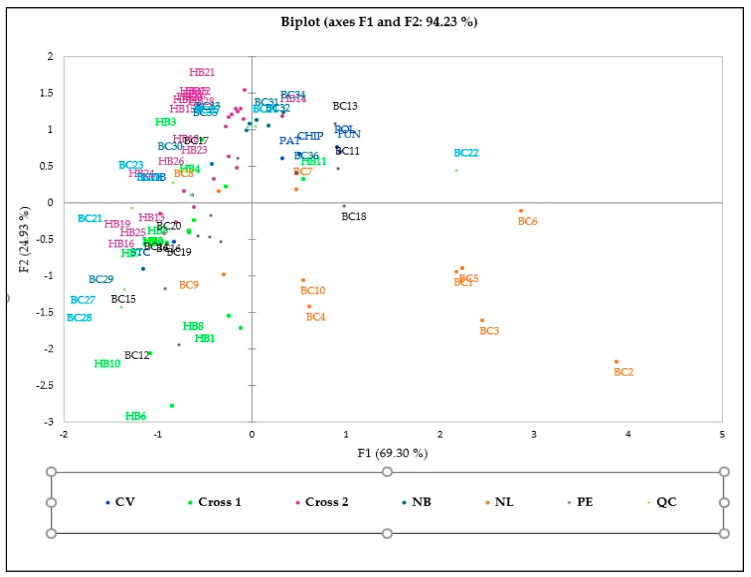
PCA biplot based on biochemical characteristics of 70 genotypes and correlation among quantitative variables.

The PCA distribution could differentiate genotypes based on biochemical characteristics, but no clear cluster was detected among the 70 genotypes (Figure 3). The biplot axes explain 94.23% of the information, out of which *y*-axes (F1) and *x*-axes account for 69.30% and 24.93%, respectively.

### 3.2. Genetic Diversity

#### 3.2.1. Analysis of Primer’s Discriminatory Capacity

For 10 EST–SSR primers, the polymorphic information content (*PIC*) values varied between 0.03 for marker CA23 to 0.96 for CA112, with an average of 0.35 (Table 4), which suggested that CA112 is the most informative and CA23 the poorest among EST–SSR primer pairs. All other EST–SSR primers, except CA787 and NA961, fell into a moderate category, with *PIC* values between 0.5 and 0.25 [38]. While markers CA236 and NA961 had the highest effective multiplex ratio (*EMR*) value (1.80), CA112 was the poorest, with an *EMR* value of 0.08. The marker index (*MI*) ranged from 0.03 to 0.65 for markers CA23 and CA421, respectively. Discrimination power (*D*) was the highest for CA236 (0.91) and the lowest for CA23 (0.03). Resolving power (*R*) ranged from 0.03 (CA23) to 2.91 (CA421). EST–SSR primer CA23 is the poorest primer pair among all primers in the category, with the lowest *MI*, *D,* and *R* values. The highest values for *MI* and *R* were observed in CA421, followed by CA236 (Table 4). The latter was, however, the best for *D* values (0.91), and it was followed by CA483 (0.90).

All eight G–SSR primer pairs fell into the moderate informative category, with *PIC* values ranging from 0.26 to 0.37, except VCC_12 and VCC_J1, which had a *PIC* value of 0.21. For G–SSR, the *EMR* values ranged from 0.63 (VCC_B3) to 2.44 (VCC_J9), and *MI* ranged from 0.18 (VCC_I2 and VCC_J1) to 0.63 (VCC_J9 and VCC_K4). A similar trend was also observed for D and R’s values, where VCC_I2 and VCC_J1 possessed the lowest values (0.27 and 0.29, respectively), and VCC_K4 acquired the highest values (0.94 and 3.11, respectively). Therefore, VCC_K4 is the most powerful primer pair, with the highest MI, D, and R values, while VCC_I2 and VCC_J1, the poorest, with the lowest values of *MI*, *D,* and *R* among G–SSR primer pairs (Table 4).

When eight EST-PCR primers were used, PIC was highest for CA227 and CA1423 (0.37) and the lowest for NA27. The *EMR* values ranged from 0.86 (CA287) to 3.57 (CA54). MI was highest for CA227 (1.16) and lowest for NA27 (0.03). The trends for *D* and *R* values were almost similar. The highest value (0.96) for *D* was observed in CA21, CA791, and CA54, while NA27 attained the lowest value (0.03). The highest value (4.31) of R was observed in CA791, followed by CA54 (4.11), while the lowest was 0.03 for NA27 (Table 4).

Among the three groups of primers, the average values of *EMR*, *MI*, *D,* and *R* suggest that the EST–PCR primer system is the most effective primer system, rather than the G–SSR and EST–SSR primer systems (Table 4).

#### 3.2.2. Analysis of Population Genetic Diversity

For EST–SSR primers, the percentage of polymorphic loci (*PL*) among the seven populations ranged from 29% for cultivars to 79% for PE clones and hybrid group Cross 2, with a mean of 62% (Table 5). The observed (*Na*) and effective number of alleles (*Ne*) were also highest in hybrid group Cross 2 (*Na* = 10.08; *Ne* = 1.48) and lowest in cultivars (*Na* = 3.75; *Ne* = 1.30). Nei’s gene diversity or expected heterozygosity (*He*) and Shannon’s information index (*I*) were also highest in the Cross 2 group (*He* = 0.28, *I* = 0.42) while *He* was the lowest in cultivars and hybrid group Cross 1 (*He* = 0.17). Hybrid group Cross 1 has the lowest value for *I* (0.25), followed by cultivars (0.26). The average values for *He* and *I* were 0.23 and 0.34, respectively (Table 5).

For G–SSR primer pairs, the *PL*, like for EST–SSRs, was highest in hybrid group Cross 2 (64%) and lowest in cultivars (32%), with an average of 51%. The number of observed alleles was the highest in hybrid group Cross 2 (8.38), followed by Cross 1 (6.25). The QC clones had the lowest number of observed alleles (4.02) and the highest number of effective alleles (*Ne* = 1.44). The average number of alleles was found to be the lowest in the cultivar (*Na* = 2.97) and PE (*Ne* = 1.28) populations. The QC clones had the highest *He* (0.24) and *I* (0.35) values, and it was followed by hybrid group Cross 2 (*He* = 0.21; *I* = 0.32). The *He* value was lowest in the Cross 1 group and PE wild clones (*He* = 0.17). Hybrid group Cross 1 also showed the lowest *I* value (0.24), followed by PE clones (0.25) (Table 5).

For the EST–PCR primer system, the values of *PL* (75.86%), *Na* (7.92), *Ne* (1.33), *He* (0.20), and *I* (0.31) were the highest for the HS2 population. The lowest level of polymorphism (46.55%) was observed in the NB population and cultivars. The number of alleles (*Na* = 2.75) was also lowest for cultivars. The effective number of alleles (*Ne* = 1.22) was minimum in the NL population. The NB population also had the lowest values of 0.14 and 0.21 for Nei’s gene diversity or expected heterozygosity (*He*) and Shannon’s information index (*I*), respectively (Table 5).

### 3.3. Cluster Analysis of Molecular Markers

#### 3.3.1. STRUCTURE Analysis

A Bayesian clustering approach was implemented to define population structures while ignoring any prior geographical information related to the sampled individuals. The STRUCTURE analysis, using EST–SSR and G–SSR markers, suggested the number of clusters to be K = 2, as indicated by the modal value of ΔK predominant peak at K = 2. For EST–SSR, while Cluster 1 was comprised of 40 genotypes, including all six cultivars (Fundy (FUN), Patriot (PAT), Chippewa (CHIP0, St. Cloud (STC), Northblue (NOB0, Polaris, (POL)) and all hybrids except HB20, Cluster 2 contained 30 genotypes, comprising hybrid HB20 and all wild clones except BC1, 10, 17, 26, 31, 33 and 34. HB20 had, however, around 48% admixture from Cluster 1. Cultivar POL clones BC10 and 33 and hybrids HB22, 23, and 27, although grouped in Cluster 1, had 28–44% admixture from Cluster 2 (Figure S1). 

With G–SSR primers, the first cluster contained two NL (BC3 and 5), five PE (BC 12, 15, 16, 19, 20), seven QC (BC 21–25, 27, 28), and six NB clones (BC 31– 36), two cultivars Fundy and Polaris, and 18 hybrids (HB 2–10, 13, 14, 19–25). The rest of the genotypes were structured into Cluster 2. While as many as eight genotypes of Cluster 1 had around 21–38% admixture from Cluster 2 (BC5, 15, 16, 21, 22, 24; POL; HB 25), in Cluster 2, hybrids HB1 and 26 and clones BC 1, 6, 9, 26 and 29 had around 30–38% admixture from Cluster 1 (Figure S2).

STRUCTURE analysis for EST–PCR primers suggests three clusters (K = 3), as indicated by the modal value of ΔK. The 70 blueberry genotypes are loosely separated, according to their phenotype, into three clusters (Figure S3). Cluster 1 consists of eight NL (BC1, 3, 5–10), seven PE (BC11–16, 18), six QC (BC21, 23–25, 27, 28) and seven NB (BC 29–34, 36) wild clones, the highbush cultivar Polaris and ten hybrids (HB13, 14, 19–26). Polaris showed an admixture of around 4% and 21% from Cluster 2 and Cluster 3, respectively. Other genotypes that showed admixture from Cluster 2 (around 15–35%) included BC7, 10 and 29 and hybrids HB19, and, from cluster 3 (around 13–38%), they were BC3, 5, 23, 28, 29, and 31. Cluster 2 consists of 13 genotypes, including four half-high cultivars (PAT, CHIP, STC, NOB), clones BC19 and 26, and hybrids HB11, 15–18, 27, and 28. In Cluster 2, the half-high cultivars St. Cloud and Patriot and hybrid HB28 showed an admixture from Cluster 1 (around 19%, 5%, and 39%, respectively) and Cluster 3 (around 19%, 38%, and 19%, respectively). In this cluster, hybrid HB16 showed an admixture of around 43% from Cluster 1. In this cluster, the lowbush wild clone BC19 showed an admixture of around 20% from Cluster 1 and 32% from Cluster 3. Cluster 3 consists of two NL (BC2, 4), two PE (BC17, 20), one QC (BC22), and one NB (BC35) wild clones, the lowbush cultivar Fundy and 11 hybrids (HB1–10, 12). Clones BC2 and BC4 had admixtures from Cluster 1 (about 29% and 41%, respectively) and Cluster 2 (about 17% and 23%, respectively). The hybrids HB1 and 12 had around 14% and 3.6%, respectively; Cluster 2, around 30%, and 32%. 

**Figure 4:**
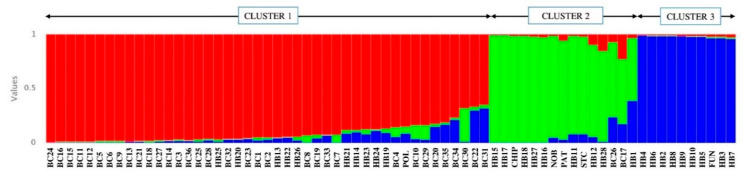
Distribution of blueberry genotypes as per STRUCTURE analysis (K = 3), based on the combination of EST–SSR, genomic (G)–SSR, and EST–PCR primer pairs. The genotypes are represented as vertical bars, and the colour represents different clusters (see Table 1 and Table 2 for genotype labels).

The combination of all three types of markers suggests three clusters (K = 3), as indicated by the modal value of ΔK in the Bayesian clustering approach. The classification was consistent with phenotypic groupings, as all wild clones except BC17 and 26 were in Cluster 1. Cluster 1 also includes the highbush cultivar Polaris and 10 hybrids (HB13, 14, 19–26). In Cluster 1, clones BC22, BC30 and BC31 showed admixtures from Cluster 2 (around 3%, 31% and 3%, respectively) and Cluster 3 (around 30%, <1% and 31%, respectively). Cluster 2 is comprised of two wild clones (BC17, 26), all four half-high cultivars (PAT, CHIP, STC, NOB), and nine hybrids (HB1, 11, 12, 15–18, 27, 28). In this cluster, while clones BC17 and 26 had admixtures from Cluster 1 (around 22% and 6%, respectively) and Cluster 3 (around 16% and 23%, respectively), hybrid HB1 showed about 3% admixture from Cluster 1 and about 38% from Cluster 3. The lowbush cultivar Fundy is grouped with nine other hybrids (BH2–10) in Cluster 3, with no significant admixture from the other groups (Figure 4).

#### 3.3.2. Unweighted Neighbour-Joining (NJ) Tree

The NJ analysis displays interindividual distances graphically. Blueberry genotypes were resolved with statistical confidence based on Jaccard’s dissimilarity coefficients [51]. As in the STRUCTURE analysis (Figure S1), two main clusters are observed for EST–SSR primer pairs (Figure S4). Cluster 1 is comprised of 39 genotypes, including one NL (BC1), one PE (BC17), one QC (BC26) and five NB (BC31, 33–36) wild clones, all lowbush and half-high cultivars (FUN, PAT, CHIP, STC, NOB) and all hybrids except HB20 and 22. Cluster 2 has 31 genotypes: nine NL (BC2–20), nine PE (BC11–16, 18–20), seven QC (BC21–25, 27, 28) and three NB (BC29, 30, 32) wild clones, highbush cultivar Polaris and two hybrids (HB20, 22). The NJ tree resembles the clusters of the STRUCTURE analysis (Figure S1), with few exceptions. The lowbush wild clones BC35 and 36, which are part of Cluster 1 of the NJ tree (Figure S4), are grouped into Cluster 2 of the STRUCTURE analysis (Figure S1). The highbush cultivar Polaris and hybrid HB22 in Cluster 2 of the NJ tree (Figure S4) are part of Cluster 1 of the STRUCTURE analysis (Figure S1).

Unlike the STRUCTURE analysis for G–SSR (Figure S2), the genotypes are grouped into three clusters in the NJ analysis (Figure S5). Cluster 1 of the NJ tree consists of two NL (BC3, 9), three PE (BC12, 19, 20), four QC (BC22, 23, 25, 27) and six NB (BC31–36) clones, lowbush cultivar Fundy and 18 hybrids (HB2–10, 13, 14, 19–24, 26). Cluster 2 possesses four NL (BC4, 7, 8,10), two PE (BC11, 17), one QC (BC26) and one NB (BC30) wild clones, three half-high cultivars (CHIP, STC, NOB) and nine hybrids (HB1, 11, 12, 15–18, 27, 28). Cluster 3 consists of four NL (B1, 2, 5, 6), five PE (BC13–16, 18), three QC (BC21, 24, 28) and one NB (BC29) clones, highbush and half-high cultivars Polaris and Patriot, and the hybrid HB25. The eight genotypes from Cluster 1 of the STRUCTURE (Figure S2) analysis (BC5, 15, 16, 21, 24, 28, POL, HB25) are part of Cluster 3 of the NJ tree (Figure S5). Similarly, genotypes BC9 and HB26, which are part of Cluster 1 of the NJ analysis (Figure S3), are part of Cluster 2 of the STRUCTURE analysis (Figure S2). The genotypes from Cluster 2 of the STRUCTURE analysis (Figure S2; BC1, 2, 6, 13, 14, 18, 29, PAT) are grouped in Cluster 3 of the NJ tree (Figure S5).

The NJ tree of EST–PCR divides the 70 genotypes into three clusters (Figure S6). Cluster 1 consists of three NL (BC1, 6, 9), eight PE (BC11–14, 15, 16, 18, 20), five QC (BC21, 23–25, 27) and six NB (BC30–34, 36) clones, highbush cultivar Polaris, and 10 hybrids (HB13, 14, 19–26). As many as four subclusters can be identified in this cluster; nine hybrids from the second cross form a subcluster with one NL (BC1), two PE clones (BC11, 16), and the cultivar Polaris. Cluster 2 contains seven NL (BC2–5, 7, 8, 10), two PE (BC17, 19) and one QC (BC26) clones, three half-high cultivars (CHIP, STC, NOB) and seven hybrids (HB1, 11, 15–18, 27). Cluster 2 is divided into three subclusters, where Northblue is grouped with five hybrids in a sub-subcluster (Figure S6). Cluster 3 consists of two QC (BC22, 28) and two NB (BC29, 35) clones, lowbush cultivar Fundy, half-high cultivar Patriot and 11 hybrids (HB2–10, 12, 28). Cluster 3 can be resolved into two main subclusters, where Fundy forms a group with nine hybrids and BC35 in a subcluster. The other two hybrids (HB12, 28) of this cluster are grouped with cultivar Patriot and clones BC22, 28, and 29 in the second subcluster (Figure S6).

**Figure 5:**
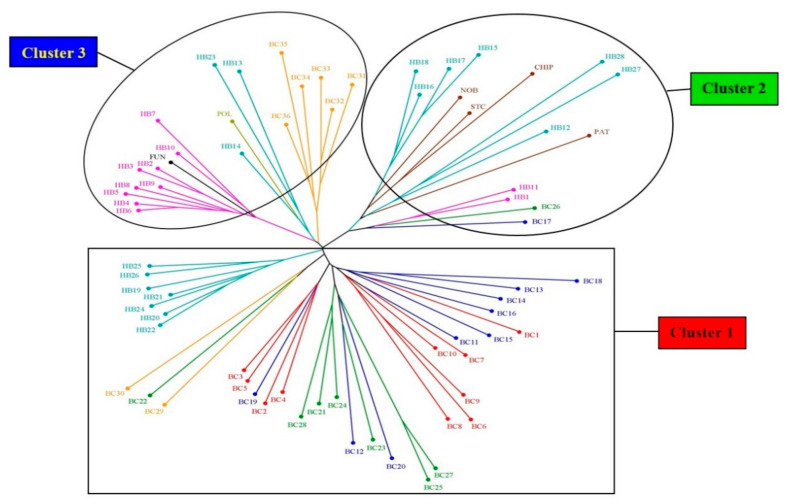
Dendrogram produced using the unweighted neighbour-joining (NJ) method based on genetic dissimilarity produced by the combination of EST–SSR, genomic (G)–SSR, and EST–PCR markers among blueberry genotypes. The colour of the branches indicates different groups (see Table 1 and Table 2 for genotype labels).

The NJ tree, generated by using all three types of markers, divides the 70 blueberry genotypes into three major clusters (Figure 5). Like Cluster 1 of the STRUCTURE analysis (Figure 4), Cluster 1 of NJ tree also consists of all 10 NL (BC1–10), nine PE (BC11–16, 18–20), seven QC (BC21–25, 27, 28) and two NB (BC29, 30) clones. Apart from these wild clones, Cluster 1 also contains seven hybrids (HB19–22, 24–26) but in an isolated subcluster (Figure 5). Cluster 2 contains PE clone BC17 and QC clone BC26, all four half-high cultivars (PAT, CHIP, STC, NOB) and nine hybrids (HB1, 11, 12, 15–18, 27, 28), collaborating the same clustering in the STRUCTURE analysis, where all these genotypes are grouped in Cluster 2 (Figure 4). Cluster 2 can be resolved into three subclusters, where Northblue, St. Cloud, and Chippewa form a subcluster with four hybrids (HB15–18), and Patriot forms another subcluster with three hybrids (HB12, 27, 28). The third subcluster of this cluster consists of hybrids HB1 and 11 and clones BC17 and 26 (Figure 5). Cluster 3 is comprised of six NB wild clones (BC31–36), cultivars Fundy and Polaris, and 12 hybrids (HB2–10, 13, 14, 23). In this cluster, three distinct subclusters are noticed: (i) six NB wild clones (BC31–36), (ii) Polaris, with three hybrids from Cross 2 (HB13, 14, 23), and (iii) Fundy, with nine hybrids from the first cross (HB2–10; Figure 5).

#### 3.3.3. Principal Coordinate Analysis (PCoA)

PCoA revealed the genetic relationship of the 70 genotypes, which was in support of the Bayesian inferences from the STRUCTURE and unweighted neighbour-joining analyses for most of the genotypes. PCoA for EST–SSR (Figure S7) confirms the STRUCTURE (Figure S1) and NJ groupings (Figure S4), as most of the lowbush wild clones are on the left side of the axis (Cluster 2), except for BC1, BC10, BC17, BC26, BC32, and BC34. All six blueberry cultivars and all hybrids, except HB20 and 22, are also placed on the right side of the axis (Cluster 1, Figure S4).

The separation of the majority of genotypes in PCoA for the G–SSR primers (Figure S8) is also aligned with the STRUCTURE (Figure S2) and NJ groupings (Figure S5). Most of the genotypes that are on the right side of the axis of the PCoA graph (Cluster 1, Figure S8) are also represented in Cluster 1 of the STRUCTURE analysis (Figure S2). The majority of these genotypes are also present in Cluster 1 of the NJ tree (Figure S5). Similarly, genotypes present on the left side of the PCoA graph are also assembled in Cluster 2 of the STRUCTURE and NJ groupings. The genotypes from Cluster 3 of the NJ tree (Figure S5) can be seen in the PCoA graph’s center, close to the central axis (Figure S8). 

The PCoA of EST–PCR primers (Figure S9) resembles the results of STRUCTURE (Figure S3) and NJ analyses (Figure S6) for the majority of the genotypes. The bottom right quadrant of the PCoA graph contains all genotypes from Cluster 1 of STRUCTURE and NJ analyses. The genotypes of Cluster 2 of STRUCTURE and NJ analyses are found in the top two quadrants of the PCoA graph. Similarly, genotypes of Cluster 3 of STRUCTURE and NJ analyses are seen, grouped together, at the far-left end of the PCoA graph. 

**Figure 6:**
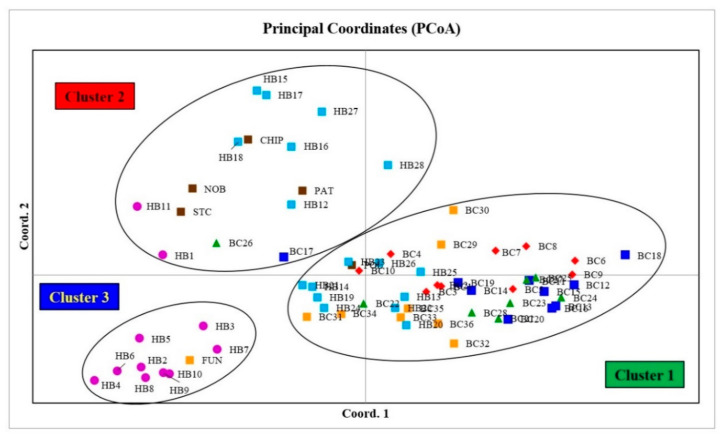
2D principle coordinate analysis (PCoA) plot of blueberry genotypes using genetic distance matrix produced by the combination of EST-SSR, genomic (G)–SSR and EST-PCR markers. The colour and shape of the points indicate different groups (see Table 1 and Table 2 for genotype labels).

The PCoA graph for combined analysis of all markers (Figure 6) also confirms the clustering patterns of the STRUCTURE (Figure 4) and NJ analyses (Figure 5) for most of the blueberry genotypes, where cultivar Fundy and nine hybrids (HB2–10) from Cluster 3 of the STRUCTURE analysis and the NJ tree can be found in the lower-left quadrant of the PCoA graph (Cluster 3; Figure 6). The individuals from Cluster 2 of the STRUCTURE analysis and the NJ tree can be found in the upper-left quadrant of the PCoA graph. Similarly, individuals from Cluster 1 of the STRUCTURE analysis and the NJ tree can be found on the right side of the central axis of the PCoA graph.

#### 3.3.4. Analysis of Molecular Variance (AMOVA)

There were significant differences among the seven groups of blueberries (*p* ≤ 0.0001), demonstrating a high genetic diversity level. This was confirmed with relatively high values for total differentiation (PhiPT: 0.228, 0.182, 0.167, and 0.186 for EST–SSR, G–SSR, EST–PCR, and combined primer pairs, respectively) for all groups, showing little similarity among them. In the present study, AMOVA analysis of EST–SSR, G–SSR, EST–PCR, and all primers combined showed a variance of 23%, 18%, 17%, and 19%, respectively, among the groups. The values for variation among genotypes within these groups were 77%, 82%, 83%, and 81%, respectively (Table 6).

### 3.4. Relationship between Biochemical and Genetic Analysis

**Figure 7:**
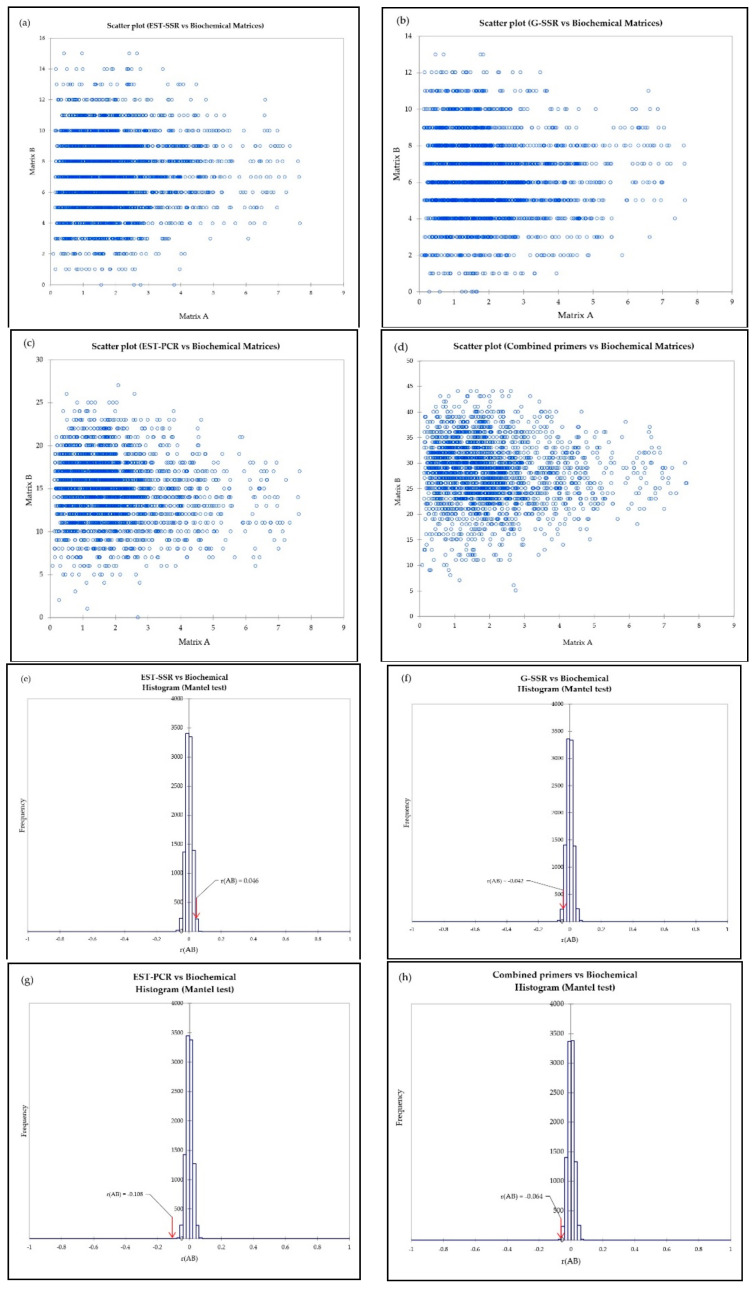
The scatter plot (**a**–**d**) and histogram (**e**–**h**) of the Mantel test between biochemical and genetic matrices (EST–SSR, G–SSR, EST–PCR and combined primers, respectively).

The Mantel test was used to check the correlation between biochemical and genetic distances of EST–SSR, G–SSR, EST–PCR, and all primers combined. Figure 7 shows there was no significant correlation between biochemical and genetic distances, as indicated by scatter plots (a, b, c, d) and poor correlation coefficient values (e, f, g, h), r(AB), of 0.046, −0.042, −0.018, and −0.064 for EST–SSR, G–SSR, EST–PCR, and combined primers, respectively.

Results of SMRA between polymorphic EST-SSR, G-SSR and EST-PCR markers, with 24, 25 and 58 alleles, respectively, and the biochemical traits in the 70 genotypes are represented in Table 7. Alleles showing significant association based on multiple correlation co-efficient (R^2^) were considered. SMRA identified 17 alleles associated with various biochemical components. According to SMRA calculations of fraction of variation for each primer pair (R^2^; Table 7), it was revealed that a combination of four alleles accounted for 33% of TAA among 70 blueberry genotypes. Out of these four, only VCC_I2_1 showed a positive, statistically significant (t = 2.343, *p* < 0.022) correlation with TAA, as explained by a beta coefficient value of 0.242. The other three alleles, VCC_S10_1, NA800_1 and CA791_4, had a statistically significant but negative correlation to TAA (Table 7). TPC variation (69%) was explained by 11 alleles, out of which seven alleles, CA1423_6, VCC_K4_6, CA54_7, CA23_1, VCC_K4_1, CA1029_1 and VCC_K4_9, were positively correlated, while other four alleles, CA54_3, VCC_I8_1, CA791_7 and CA21_7, were negatively correlated with TPC (Table 7). VCC_K4_6 was the highest contributor, with a beta coefficient value of 0.585, and positively significant (t = 5.554, *p* < 0.000). Six alleles, VCC_K4_6, CA54_7, CA23_1, CA1029_1, VCC_K4_1 and CA1423_1, out of seven alleles were positively correlated with TFC. Allele CA791_1 was negatively correlated with TFC. VCC_K4_6 (t = 8.369, *p* < 0.000) was the key contributor, with a proportion of variation of 21% and a beta coefficient value of 0.818 (Table 7). Five alleles, VCC_K4_6, CA54_7, CA23_1, VCC_K4_1 and CA1029_1, were also associated and positively correlated with TPC as well as TFC (Table 7).

## 4. Discussion

The study presented here provides insight into genetic diversity, with respect to genetic relationship and structure, and biochemical properties of two groups of selected hybrids of lowbush and half-high blueberries, half-high wild blueberry clones, and highbush and lowbush blueberry cultivars. The antioxidant properties of blueberries are well known for their medicinal value in negating the harmful effects of free radicals [56]. The leaves of blueberry wild clones and cultivars can have higher antioxidant activity [57,58], polyphenolics, and proanthocyanidins than the fruit [59,60]. 

The antioxidant activity depends on the synergistic and antagonistic interaction of various compounds and environmental factors [61]. There is no standard agreed method for estimating antioxidant activity because of its complexity [62]. In the present study, we used the DPPH radical scavenging method, as it is sensitive and cheaper than other known procedures [63]. Out of all the groups, the NB wild clones had the highest TAA, followed by CV, Cross 2, and NL wild clones. TPC and TFC were highest in NL wild clones, followed by CV. The wild clones from NL and NB proved to be an important resource for improving antioxidant properties in the blueberry breeding program. Phenolics are the abundantly available secondary metabolite derived from phenylalanine via the secondary metabolic pathway, catalyzed by phenylalanine lyase L (PAL). Various biotic and abiotic factors can cause stress in source plants and trigger higher activity of PAL [64]. Low levels of light in the NL province could have contributed to higher levels of TAA, TPC, and TFC. Leaf maturity can have a significant impact on phytochemical composition in blueberry. In their study, Riihinen et al. [60] reported the red leaves of *V. corymbosum* possessed higher levels of quercetin and kaempferol, p-coumaric, and caffeic acids than the green leaves. This could be the case because solar radiation increases these compounds as a part of the photoprotective mechanism [60]. On top of that, the red leaves contained a small amount of anthocyanins, while green leaves did not have any anthocyanin content [65]. Therefore, TPC and TFC may not sufficiently explain total antioxidant activity as they are the cocktail of various compounds and their activities. The DPPH value is calculated by the addition of various antioxidant compounds, which depends on the chemical used during the extraction of leaves [66]. However, Wang and Lin [67] reported contradicting observation that the young leaves from different varieties of blackberries, raspberries and strawberries possessed higher TPC and TAA than older leaves. There was a positive correlation between TAA with TPC and TFC, which was also reported in previous studies involving blueberries [28,29,68].

The biochemical analysis in the present study provides important information about the diversity of antioxidant properties. However, biochemical characteristics by themselves are not enough for the presence of genetic diversity. The DNA marker system provides a precise and reliable method for further analysis of variability. The extent of genetic diversity between and within populations is often the outcome of a combination of factors such as gene flow, genetic drift, inbreeding, mutation and the selection effect [43]. It is very expensive, time-consuming, and laborious to develop species-specific molecular markers. Because of these constraints, we used EST–SSR, G–SSR, and EST–PCR markers developed for highbush blueberries [19,20]. Our report is apparently the first to use these three types of markers to assess genetic diversity in a group of hybrids obtained by crossing lowbush with half-high blueberries. Microsatellite markers have also been used for hybrid identification in closely related wild *Petunia* species [69]. Although G–SSR markers are highly abundant in the plant genome and are attractive due to their reproducibility and polymorphic nature, most of them lack close linkage to transcribed regions and do not have a specific genic function. On the other hand, SSR markers derived from EST sequences are associated with the genome’s transcribed or expressed regions [70]. The single-pass sequence of cDNA clones that are picked randomly is the source of EST–SSR and EST–PCR markers [71]. All primer pairs used in this study showed an elevated polymorphism that confirmed the high degree of genetic diversity in the blueberry genome of the current material.

In the present study, the discriminatory power of EST–SSR, G–SSR, and EST–PCR primer pairs was compared by *PIC*, *EMR*, *MI, D,* and *R*. These values help in determining the effectiveness of a specific primer pair in the analysis of genetic diversity. Although *PIC* values for EST–SSR (average 0.35) and G–SSR primer pairs (average 0.30) were higher than EST–PCR (average 0.28), *EMR*, *MI, D,* and *R* values for EST-PCR markers were the highest (average 2.05, 0.61, 0.79 and 2.37, respectively) followed by G–SSR (average 1.28, 0.38, 0.65 and 1.08, respectively) and EST–SSR (average 1.13, 0.33, 0.54 and 1.02, respectively). The highest *PIC* value for EST-SSR primer pair CA112 (0.96), combined with very low values for *EMR*, *MI, D* and *R* (0.08 for *EMR*, *MI*, *D* and 0.09 for *R*; Table 4), proved that this primer pair is not worthy for analyzing present blueberry hybrids, wild clones, and cultivars. On the other hand, the EST-PCR primer pair CA227, with its highest *MI* value among all primers (1.16), was the best for overall utility to study the present material, and it was followed by CA1423 (*MI* = 1.00) and CA54 (*MI* = 0.99). These three EST-PCR primer pairs may be very valuable for analyzing blueberry hybrids. However, the moderate-to-high values for most of the primer pairs could be attributed to their effectiveness in studying the genetic diversity of the present material.

In the present study, the mean allele number for EST–SSR, G–SSR and EST–PCR were 6.30, 5.36 and 4.40, respectively, which is comparable or lower than previous studies involving SSR and/or EST–PCR primer pairs in blueberries (22.4; [72]; 4.8; [21]; 18.5; [73]; 8.33; [16]; 17; [10]; 14.24; [18]; 10; [74]; 20.5; [22]; 14.33; [17]). The average Shannon’s index (*I*) of 0.34 for EST–SSR, 0.29 for G–SSR and 0.26 for EST–PCR are lower than those recorded for *V. vitis-idaea* (0.57; [75]) and *V. myrtillus* (0.55; [13]), *V. uliginosum* (0.65; [76]), *V. corymbosum* (0.62; [18]) and *Vaccinium* species (1.93; [10]; 2.56; [22]). Average *He* values for EST–SSR (0.23), G–SSR (0.19) and EST-PCR (0.16) were also less than those reported in previous studies with blueberries (0.88; [73]; 0.81; [74]; 0.87; [18]; 0.86; [10]; 0.80; [22]; 0.56; [17]. The lower values of diversity parameter could be an indication of genetic erosion resulting from selective farming and deforestation [3].

We used three complementary methods: STRUCTURE, NJ tree, and PCoA to study population structure and genotype relationships in wild, cultivated, and hybrid blueberries using 26 PCR-based marker pairs. Genotype identification using DNA markers is favoured due to their consistency and reliability, as they are unaffected by the environment [77]. The combined STRUCTURE analysis divided the genotypes into three major groups, with some admixtures confirmed by PCoA and NJ analyses for most genotypes. Admixtures in the wild blueberry clones that were observed in the present material with STRUCTURE analysis might be due to the consequence of a glacial bottleneck and quick colonization of these blueberries, along with increased regional gene flow due to the migration of human beings and trade in agriculture [78]. Although the hybrids were distributed in all three clusters, most of them formed distinct subgroups, either alone or with lowbush or half-high cultivars. This might be because they had been developed through crossing between lowbush and half-high blueberries and share the genes from both parents. However, most of the wild lowbush blueberries, except for the NB clones and the half-high cultivars, were grouped based on their phenotypes. While lowbush blueberries are less than 0.5 m tall, the suckering to crown-forming of half-high blueberry plants are 0.5 to 1.0 m tall. Highbush blueberry plants are crown-forming and 2.0 or higher in height [79]. In the present study, most of the wild clones, although collected from four different provinces, did not group based on their collection place. Although there is a wide genetic variation among the wild clones, there is no pattern of differentiation based on their collection places. This was also observed in AMOVA analysis, where in the combined analysis, the variation among groups was 19%, and most of the variations (81%) were among the genotypes within provinces, cultivars or hybrid groups. Similar observations were also reported by Debnath [10] and Tailor, Bykova, Igamberdiev and Debnath [22], who worked with different sets of wild lowbush blueberry clones and observed that wild clones were grouped into different clusters. In the present study, it was evident that there was no clear difference between the wild, cultivated, and hybrid blueberries, indicating that diversity-wise, the present genotypes are all heterogeneous in nature. This might be due to a smaller variation among different groups than the variation among the genotypes within a group. STRUCTURE, NJ and PCoA analyses, along with AMOVA analysis, were complementary to each other and, thus, instead of using one method, a number of procedures are more informative for drawing valid conclusions. Similarly, using more than one type of molecular marker is always better than using a single type of molecular marker [10,22]. In our study, STRUCTURE, NJ, PCoA and AMOVA analyses using EST–SSR, G–SSR and ESTPCR markers have well discriminated the wild blueberry clones, cultivars and hybrids from the wild and cultivated blueberries that are part of our current germplasm repository for the cool climates of Canada.

There are no reports available on the relationship between molecular markers and biochemical properties in blueberry. Our study found no parallels between genetic and biochemical data, as observed by phylogenetic trees, PCA–PCoA graphs, and the Mantel test of correlation. The poor correlation between genetic clustering from biochemical data indicates varying genomic coverage in blueberries. Molecular markers span across the genome and most of which are not expressed at the phenotypic level. The noncoding regions of the genome that are not accessible to phenotypic expression might be the reason for the dissimilarity between molecular and chemical diversity [77]. There are only three reports available on the comparative analysis of molecular markers with biochemical properties. In their study, Debnath and Sion [80] reported no correlation between genetic diversity based on ISSR markers and chemical diversity based on antioxidant activity and anthocyanin content in lingonberry. Similar observations were also reported in strawberry [81] and cranberry using ISSR, EST–SSR, and EST–PCR markers [82]. We also studied the association of EST–SSR, G–SSR and EST–PCR markers with 70 genotypes of blueberry and found that only one marker was associated with TPC, as revealed by DPPH assay, and five markers were associated with both TPC and TFC. This can be explained by the polyploid nature of blueberry and the distribution of associated alleles across the whole genome. However, our study is the first one to use SMRA to identify markers associated with antioxidant properties. This method can provide easy and reliable identification of favourable genotypes or populations in a breeding program at an early stage and has been used to associate molecular markers with traits in numerous species such as mulberry [83], buckthorn [84], and Tunisian olive [85]. This approach is a convenient and quick tool for marker–trait association, without the need to map populations. Multigenic control of TPC, TFC and antioxidant traits can have practical uses in future blueberry breeding programs.

Blueberries are of significant importance for their antioxidant phytochemicals, especially phenolic metabolites that play a significant role in human health benefits and plant defence mechanisms [86]. Most of plant phenolics are flavonoids and nonflavonoids. Flavonoids are of two types: anthocyanins and anthoxanthins. While anthocyanins are pigment molecules (red, blue and purple), anthoxanthins are white to yellow or colourless molecules and include flavanols, flavonols, flavones and isoflavones. Nonflavonoids are comprised of phenolic acids, lignans and stilbenes. Tannin and lignin are the other nonflavonoid subclasses [86]. The flavonol quercetin is an important nutritional bioactive compound with high bioaccessibility (~80%) [87]. Quercetin helps in protecting against osteoporosis, cancer, pulmonary and cardiovascular diseases, and ageing [88]. In blueberry, anthocyanins were found to possess the highest inhibition effects on in vitro colon cancer cell proliferation, followed by flavonols and tannins [89]. Biomarker-based human clinical studies showed that regular and moderate consumption of blueberries and/or anthocyanins is associated with reduced risk of death, cardiovascular disease and type 2 diabetes [90]. In another study with in vitro cell bioassays for anti-inflammatory and antioxidant activities, Grace et al. [91] reported that the anthocyanin group of phenolics was mainly responsible for the bioactivity, and blueberry extract suppressed proinflammatory markers (in-terleukin-1β, cyclooxygenase-2, inducible nitric oxidesynthase, and interleukin-6 [92]). Polyphenol-, anthocyanin- and proanthocyanidin-rich components of crude wild blueberry extract were found to suppress mRNA biomarkers of acute inflammation, and mlvidin-3-glucoside suppressed the effects of proinflammatory genes that are responsible for transcriptional regulation and cytokine-mediated inflammation [92]. It has been observed that in-vitro antioxidant assays with blueberries resulted in a strong correlation with those of total phenolic and total anthocyanin contents [91]. In the current study, we measured total phenolic, flavonoid and antioxidant contents to study genetic and biochemical diversity in blueberry germplasm and to identify blueberry genotypes with high bioactive components and wide diversity for use in an on-going breeding program. Identifying phenolic-rich cultivars for breeding species with high bioactive composition is an important approach to improving the nutritional quality of blueberries. Crossing between selected genotypes is expected to develop new cultivars that combine superior health-promoting bioactive components with diverse adaptability under a changing environment. However, blueberry genotypes with more specific profiles of polyphenols are of significant importance for human health, which can be explored in future research with some of the selected promising genotypes from the current material. As to the mass balance of phenolics and what is in human blood, the amount observed is low, especially for highly hydrophilic phenolics. In this, researchers have earlier ignored the metabolites that are also present; if this was done appropriately, the actual intake is much higher than was originally thought. Efforts have also been made to lipophilize phenolic compounds to enhance their absorption. Therefore, having more phenolics, especially those with different polarities, is a good idea, as a mixture of phenolics is present in each material (personal communication: F. Shahidi). Valuable single phenolic compounds can be estimated with the selected material after chromatographic separation, as the total assays do not reflect the situation in terms of polyphenols; they also can target amino acids and reducing agents.

However, when dealing with the same type of material, the trends provided are quite informative, and although absolute values may not be exact, the trends are always valid. This assumes that the amino acids/proteins present are not varied to any great extent, which is a valid assumption in almost all cases (personal communication: F. Shahidi).

## 5. Conclusions

Our study is the first of its kind to investigate antioxidant activity and phenolic and flavonoid contents, along with genetic diversity analysis, using three types of marker systems—EST–SSR, G–SSR, and EST–PCR—in blueberry. The study identified two NL (BC2, 6) for TAA and one QC wild clone (BC22) for TPC and TFC, superior to cultivars and hybrids. These wild blueberry clones hold the key to designing future breeding exercises to generate cultivars with valuable antioxidant traits. The present study indicates that 10 EST–SSR, eight G–SSR, and eight EST–PCR primer pairs could distinguish and report genetic variations at the molecular level among wild and cultivated lowbush, half-high and highbush blueberries and among hybrids between lowbush and half-high blueberries. The EST–PCR primer pair CA227 was the best to discriminate blueberry hybrids, clones, and cultivars, followed by EST–PCR primer pairs CA1423 and CA54. The alleles of CA1423 and CA54 also showed a strong positive correlation and association to TPC and TFC in SMRA. The utility of these primers across different blueberry species can help identify and characterize interspecies blueberry hybrids and select useful genotypes as a parent in a breeding program. DNA fingerprinting with more than one type of molecular marker will allow better management of the blueberry germplasm and conservation efforts. Clustering based on EST–SSR, G–SSR, EST–PCR, and combined primer data was different from antioxidant properties. These markers are spread across the genome, many of which are located in noncoding regions, explaining the poor correlation between genetic and biochemical data. However, these markers’ potential utility is immense, as shown by our association study in the blueberry marker–biochemical relationship using SMRA, and can prove to be a valuable tool.

## Figures and Tables

**Table 1:** Parentage of lowbush, half-high, and highbush blueberry cultivars and hybrids.

Cultivar/Hybrid	Parentage
Lowbush	
Fundy	An open-pollinated seedling of cultivar Augusta [24]
Half-high	
Patriot	US3 (Dixi × Michigan lowbush No. 1) × Earliblue [25]
Chippewa	B18A (G65 × Ashworth) × US3 (Dixi × Michigan lowbush No. 1) [26]
St. Cloud	B19 (G65 × Ashworth) × US3 (Dixi × Michigan lowbush No. 1) [25]
Northblue	B10 (G65 × Ashworth) × US3 (Dixi × Michigan lowbush No. 1) [27]
Highbush	
Polaris (POL)	B15 (G65 × Ashworth) × Bluetta [26]
Hybrids	
Cross 1 (HB1–11)	Wild clone × Half-high/highbush cultivar (Debnath, personal communication)
Cross 2 (HB12–28)	Wild clone × Half-high/highbush cultivar (Debnath, personal communication)

**Table 2:** Wild blueberry clones, designated as BC1 to BC36, collected from the Canadian provinces of Newfoundland and Labrador (NL), Prince Edward Island (PE), Quebec (QC), and New Brunswick (NB).

Clone	No. of Clones	Province	Community	Latitude (N)	Longitude (W)
BC1–9	6	NL	North River	47°32′	53°18′
BC6–8, 10	4	NL	Sears town	47°35′	53°17′
BC11–20	10	PE	Blooming Point	46°23′	62°58′
BC21, 27, 28	3	QC	Longue-River	48°33′	69°14′
BC22, 23, 26	3	QC	Baie-Trinite	49°25′	67°18′
BC24	1	QC	Pointe-Lebel	49°09′	68°13′
BC25	1	QC	Baie-Comeau	49°13′	68°08′
BC29–36	8	NB	Little Shemogue	46°06′	64°01′
Total	36				

**Table 3:** Total antioxidant activity (TAA), phenolic (TPC) and flavonoid contents (TFC) of groups of wild clones collected from four Canadian provinces: Newfoundland and Labrador (NL), Prince Edward Island (PE), Quebec (QC), and New Brunswick (NB). Cultivars (CV): Fundy, Polaris, Patriot, Chippewa, St. Cloud, Northblue (NOB); hybrids: Cross 1 and Cross 2. GAE = gallic acid equivalent; CE = catechin equivalent; fl = fresh leaf.

Clones/Cultivars/Hybrids (No.)		TAA (mg GAE/g fl)	TPC (mg GAE /g of fl)	TFC (mg CE/g fl)
NL (10)	Mean ± SD	3.94 ± 1.02 ab	0.16 ± 0.05 b	5.26 ± 2.49 b
	Min–Max (Fold)	2.45–5.82 (2.37)	0.09–0.24 (2.60)	2.00–9.99 (4.99)
PE (10)	Mean ± SD	3.34 ± 1.36 ab	0.12 ± 0.02 ab	2.14 ± 0.79 a
	Min–Max (Fold)	1.14–5.45 (4.77)	0.09–0.15 (1.58)	1.22–3.77 (3.09)
QC (8)	Mean ± SD	3.35 ± 1.64 ab	0.09 ± 0.04 a	1.88 ± 1.59 a
	Min–Max (Fold)	1.14–5.82 (5.09)	0.06–0.18 (2.79)	0.81–5.56 (6.85)
NB (8)	Mean ± SD	4.29 ± 1.06 a	0.11 ± 0.02 ab	1.74 ± 0.46 a
	Min–Max (Fold)	1.84–5.13 (2.79)	0.08–0.15 (1.95)	1.19–2.70 (2.26)
CV (6)	Mean ± SD	4.23 ± 1.10 ab	0.13 ± 0.03 ab	1.84 ± 0.84 a
	Min–Max (Fold)	2.48–5.19 (2.09)	0.09–0.16 (1.78)	0.64–2.83 (4.43)
Cross 1 (11)	Mean ± SD	2.55 ± 1.28 b	0.11 ± 0.03 ab	1.39 ± 0.18 a
	Min–Max (Fold)	0.29–4.48 (15.34)	0.07–0.16 (2.17)	1.04–1.71 (1.64)
Cross 2 (17)	Mean ± SD	4.08 ± 0.89 ab	0.10 ± 0.01 a	1.41 ± 0.29 a
	Min–Max (Fold)	2.52–5.13 (2.04)	0.08–0.12 (1.51)	0.87–1.95 (2.24)
All (70)	Mean ± SD	3.67± 0.25	0.11 ± 0.03	2.19 ± 1.69
	Min–Max (Fold)	0.29–5.82 (19.93)	0.06–0.24 (3.83)	0.64–9.99 (15.63)

Values are means ± SD values of at least three replicates. Means followed by same letter are not significantly different according to Tukey’s range test at *p* = 0.05.

**Table 4:** Discriminatory power indices of EST–SSR, genomic (G)–SSR and EST–PCR markers for diversity analysis of blueberry hybrids, wild clones and cultivars.

Primers	*PIC*	*EMR*	*MI*	*D*	*R*
EST–SSR					
CA23	0.03	0.99	0.03	0.03	0.03
CA112	0.96	0.08	0.08	0.08	0.09
CA169	0.37	1.11	0.41	0.69	0.97
CA236	0.33	1.80	0.60	0.91	2.17
CA421	0.37	1.74	0.65	0.81	2.91
CA483	0.34	0.96	0.33	0.90	1.40
CA787	0.20	0.87	0.17	0.24	0.26
NA800	0.37	1.10	0.41	0.70	1.57
NA961	0.16	1.80	0.29	0.19	0.40
NA1040	0.37	0.86	0.32	0.82	0.40
Mean	0.35	1.13	0.33	0.54	1.02
G–SSR					
VCC_I2	0.21	0.86	0.18	0.27	0.29
VCC_B3	0.34	0.63	0.21	0.90	0.86
VCC_I8	0.37	1.01	0.38	0.74	0.31
VCC_J1	0.21	0.86	0.18	0.27	0.29
VCC_J3	0.36	0.79	0.29	0.85	0.49
VCC_J9	0.26	2.44	0.63	0.34	1.11
VCC_K4	0.30	2.13	0.63	0.94	3.11
VCC_S10	0.33	1.53	0.51	0.91	2.20
Mean	0.30	1.28	0.38	0.65	1.08
EST–PCR					
CA21	0.28	1.47	0.41	0.96	2.94
CA54	0.28	3.57	0.99	0.96	4.11
CA227	0.37	3.11	1.16	0.80	3.03
CA287	0.32	0.86	0.28	0.92	0.74
CA791	0.27	2.16	0.57	0.96	4.31
CA1029	0.29	1.56	0.45	0.95	1.80
CA1423	0.37	2.69	1.00	0.71	2.00
NA27	0.03	0.99	0.03	0.03	0.03
Mean	0.28	2.05	0.61	0.79	2.37

PIC, polymorphic information content; EMR, effective multiplex ratio; MI, marker index; D, discrimination power; R, resolving power.

**Table 5:** Population genetic diversity parameters of blueberry hybrid groups (Cross 1: HB1–11; Cross 2: HB12–28), wild clones collected from Canadian provinces Newfoundland and Labrador (NL), Prince Edward Island (PE), Quebec (QC), and New Brunswick (NB), and six cultivars (CVs).

	Sample Size	*PL (%)*	*Na*	*Ne*	*He*	*I*
EST–SSR						
Cross 1	11	46	7.56	1.30	0.17	0.25
Cross 2	17	79	10.08	1.48	0.28	0.42
NL	10	71	6.44	1.48	0.27	0.40
PE	10	79	6.42	1.44	0.26	0.39
QC	8	71	4.99	1.42	0.24	0.35
NB	8	58	4.89	1.42	0.23	0.34
CV	6	29	3.75	1.30	0.17	0.26
Mean		62	6.30	1.41	0.23	0.34
G–SSR						
Cross 1	11	44	6.25	1.30	0.17	0.24
Cross 2	17	64	8.38	1.37	0.21	0.32
NL	10	60	5.64	1.29	0.18	0.28
PE	10	48	5.75	1.28	0.17	0.25
QC	8	60	4.02	1.44	0.24	0.35
NB	8	48	4.50	1.38	0.20	0.29
CV	4	32	2.97	1.31	0.18	0.27
Mean		51	5.36	1.34	0.19	0.29
EST–PCR						
Cross 1	11	50	5.06	1.28	0.16	0.24
Cross 2	17	76	7.92	1.33	0.20	0.31
NL	10	53	4.20	1.22	0.14	0.22
PE	10	66	4.22	1.27	0.17	0.27
QC	8	55	3.53	1.26	0.16	0.25
NB	8	47	3.15	1.23	0.14	0.21
CV	4	47	2.75	1.28	0.18	0.28
Mean		56	4.40	1.27	0.16	0.26

PL, percentage of polymorphic loci; Na, number of alleles; Ne, effective number of alleles; He, Nei’s genetic diversity or expected heterozygosity; I, Shannon’s information index.

**Table 6:** Genetic differentiation among blueberry genotypes by analysis of molecular variance (AMOVA) based on seven groups, where four groups were comprised of wild clones collected from four Canadian provinces, the fifth group had all six cultivars, the sixth group contained 11 hybrids from the first cross (HB1–11), and the seventh group contained 17 hybrids from the second cross (HB12–28).

Source of Variation	Marker Type	Degrees of Freedom	Sum of Squares	Mean Square	Variance Components	Percentage of Variation
Among groups	EST–SSR	6	67.133	11.189	0.847	23%
	G-SSR	6	50.291	8.382	0.586	18%
	EST–PCR	6	111.626	18.604	1.256	17%
	Combined	6	229.051	38.175	2.689	19%
Within the groups	EST–SSR	63	180.681	2.868	2.868	77%
	G–SSR	63	165.437	2.626	2.626	82%
	EST–PCR	63	394.688	6.265	6.265	83%
	Combined	63	740.806	11.759	11.759	81%
Total	EST–SSR	69	247.814		3.715	100%
	G–SSR	69	215.729		3.212	100%
	EST-PCR	69	503.314		7.521	100%
	Combined	69	969.857		14.448	100%
Stat (PhiPT)	Value	*p*				
EST-SSR	0.228	0.0001				
G-SSR	0.182					
EST-PCR	0.167					
Combined	0.186					

**Table 7:** Association of EST–SSR, G–SSR and EST–PCR markers with biochemical components in blueberry leaf extract, as revealed by stepwise multiple regression analysis (SMRA).

Traits	Alleles	Type of Marker	R	R^2^	R^2^ Change	F Change	Standardized Beta Coefficients	t Value	*p* Value
**TAA**	VCC_S10_1	G–SSR	0.378	0.143	0.143	11.363	−0.493	−4.659	0.000
	+ NA800_1	EST–SSR	0.455	0.207	0.064	5.428	−0.288	−2.757	0.008
	+ CA791_4	EST–PCR	0.525	0.276	0.068	6.225	−0.283	−2.774	0.007
	+ VCC_I2_1	G–SSR	0.576	0.332	0.056	5.490	0.242	2.343	0.022
**TPC**	CA1423_6	EST–PCR	0.335	0.112	0.112	8.585	0.281	3.563	0.001
	+ VCC_K4_6	G–SSR	0.472	0.223	0.111	9.576	0.585	5.554	0.000
	+ CA54_3	EST–PCR	0.549	0.302	0.078	7.405	−0.189	−2.372	0.021
	+ CA54_7	EST–PCR	0.603	0.363	0.062	6.309	0.310	3.878	0.000
	+ CA23_1	EST–SSR	0.652	0.425	0.061	6.826	0.368	3.519	0.001
	+ VCC_K4_1	G–SSR	0.697	0.485	0.061	7.444	0.253	3.370	0.001
	+ VCC_I8_1	G–SSR	0.727	0.529	0.043	5.670	−0.279	−3.535	0.001
	+ CA791_7	EST–PCR	0.757	0.573	0.045	6.380	−0.244	−3.253	0.002
	+ CA21_7	EST–PCR	0.785	0.617	0.043	6.805	−0.225	−2.829	0.006
	+ CA1029_1	EST–PCR	0.816	0.665	0.048	8.519	0.251	3.266	0.002
	+ VCC_K4_9	G–SSR	0.830	0.689	0.024	4.414	0.172	2.101	0.040
**TFC**	VCC_K4_6	G–SSR	0.461	0.212	0.212	18.340	0.818	8.369	0.000
	+ CA54_7	EST–PCR	0.583	0.340	0.127	12.893	0.378	5.434	0.000
	+ CA23_1	EST–SSR	0.667	0.445	0.105	12.476	0.455	4.662	0.000
	+ CA1029_1	EST–PCR	0.738	0.544	0.100	14.211	0.453	5.831	0.000
	+ VCC_K4_1	G–SSR	0.797	0.635	0.091	15.917	0.305	4.385	0.000
	+ CA791_1	EST–PCR	0.822	0.676	0.041	7.917	−0.269	−3.415	0.001
	+ CA1423_1	EST–PCR	0.837	0.700	0.025	5.116	0.165	2.262	0.027

+ Denotes the inclusion of alleles (s) in the preceding step(s) in the SMRA.

## Data Availability

Data is contained within the article and supplementary material.

## Supplementary Materials

The following are available online at https://www.mdpi.com/2076-3921/10/3/458/s1, Tables S1–S4; Figures S1–S9.
