# Supplementary material for: Genetic Diversity of Blueberry Genotypes Estimated by Antioxidant Properties and Molecular Markers
Source: Antioxidants (Basel). 2021 Mar 15;10(3):458. doi: 10.3390/antiox10030458 (PMC8001406; doi:10.3390/antiox10030458)
Supplement: Supplementary file 1 [file antioxidants-10-00458-s001.pdf]

antioxidants

MDPI

Supplementary Materials

Table S1. EST-SSR, genomic (G-) SSR and EST-PCR primer pairs: their sequences and annealing temperatures.

Primer pair[1]	Forward and Reverse primer sequence (5′-3′)	Annealing temperature (°C)
EST-SSR		
CA23	F: GAGAGGGTTTCGAGGAGGAG<br>R: GTTTAGAAACGGGACTGTGAGACG	60
CA112	F: TCCACCCACTTCACAGTTCA<br>R: GTTTATTGGGAGGGAATTGGAAAC	49
CA169	F: TAGTGGAGGGTTTTGCTTGG<br>R: GTTTATCGAAGCGAAGGTCAAAGA	52
CA236	F: GTTAAGCTTTTAGATGAGTTGATGG<br>R: GTTTAACCAGTCCCAGACCCAAAT	54
CA421	F: TCAAATTCAAAGCTCAAAATCAA<br>R: GTTTAAGGATGATCCCGAAGCTCT	58
CA483	F: GTCTTCCTCAGGTTCGGTTG<br>R: GAACGGCTCCGAAGACAG	58
CA787	F: TCCTCGTTCTCTCCCTCTCA<br>R: GTTTCGCTGAAGTTGGAGTCCTT	60
NA800	F: CAATCCATTCCAAGCATGTG<br>R: GTTTCCCTAGACCAGTGCCACTTA	62
NA961	F: TCAGACATGATTGGGGAGGT<br>R: GTTTGGAATAATAGAGGCGGTGGA	56
NA1040	F: GCAACTCCCAGACTTTCTCC<br>R: GTTTAGTCAGCAGGGTGCACAA	58
G-SSR		
VCC_B3	F: CCTTCGATCTTGTTCCTTGC<br>R: GTTTGATGCAATTGAGGTGGAGA	62
VCC_I2	F: AGGCGTTTTTGAGGCTAACA<br>R: TAAAAGTTCGGCTCGTTTGC	62
VCC_I8	F: TTCAGCATTCAATCCATCCA<br>R: GTTTCTCTTCTCCAATCTCTTTTCCA	58
VCC_J1	F: CTCATGGGTTCCCATAGACAA<br>R: TGCAGTGAGGCAAAAGATTG	60
VCC_J3	F: TGATTACATTGCCAGGGTCA<br>R: TGGAAACAACCGGGTTACAT	58
VCC_J9	F: GCGAAGAACTTCCGTCAAAA<br>R: GTGAGGGCACAAAGCTCTC	62
VCC_K4	F: CCTCCACCCCACTTTCATTA	62

	R: GCACACAGGTCCAGTTTTTG	
VCC_ S10	F: ATTTGGTGTGAAACCCCTGA	60
	R: GTTTGCGGCTATATCCGTGTTTGT	
EST-PCR		
CA21	F: TCCGATAACCGTTACCAAGC	52
	R: TATACAGCGACACGCCAAAA	
CA54	F: CCGGTGAACTTCCACTTGTT	52
	R: AGATACTACTGGGGGTGGGG	
CA227	F: TGGAGACTGGAGTGATGCAA	56
	R: TTTGCAAGAACCATGCTGAG	
CA287	F: AGGGCTTTCCCTCAATCACT	58
	R: CCTTGTTGTTCCTTCCTTCG	
CA791	F: AGAGCCAAAAGAAGGGGAAG	56
	R: TCAAAATTTTCCGGACCAG	
CA1029	F: GAAGTTTTCCGTTCTCTGCAA	52
	R: CTGCAGCTAGGACCGAAGAG	
CA1423	F: TCATAGCCAATACACTCGAACC	60
	R: GCCCCACCTTTAGCAAACTC	
NA27	F: CGCTCGCTCCATTGTTTC	60
	R: TATGCATGAAGCTTGCCGTA	

[1]Above markers were developed from EST libraries generated from floral buds of cold acclimated (designated as prefix CA) and non-acclimated (designated as prefix NA) highbush blueberry plants [20].

Table S2. Total antioxidant activity (TAA), phenolic (TPC) and flavonoid contents (TFC) of individual wild clones collected from four Canadian provinces: Newfoundland and Labrador (NL), Prince Edward Island (PE), Quebec (QC) and New Brunswick (NB), cultivars (CV): Fundy, Polaris, Patriot, Chippewa, St. Cloud, Northblue (NOB), hybrids: Cross1 and Cross2. GAE = gallic acid equivalents, CE = catechin equivalent, fl = fresh leaf.

Clones/Cultivars/Hybrids	TAA (mg GAE/g fl)	TPC (mg GAE /g of fl)	TFC (mg CE/g fl)
BC1	4.45 ± 0.07	0.18 ± 0.01	6.55 ± 0.39
BC2	4.58 ± 0.07	0.24 ± 0.01	9.99 ± 0.11
BC3	4.03 ± 0.09	0.20 ± 0.02	6.99 ± 0.12
BC4	2.75 ± 0.08	0.14 ± 0.01	3.93 ± 0.13
BC5	4.57 ± 0.05	0.20 ± 0.02	6.03 ± 0.02
BC6	5.82 ± 0.03	0.20 ± 0.01	7.07 ± 0.05
BC7	4.20 ± 0.07	0.11 ± 0.00	3.49 ± 0.03
BC8	3.53 ± 0.12	0.09 ± 0.01	2.00 ± 0.01
BC9	2.45 ± 0.07	0.11 ± 0.01	2.41 ± 0.07
BC10	3.04 ± 0.08	0.13 ± 0.00	4.18 ± 0.01
BC11	4.88 ± 0.05	0.15 ± 0.01	2.92 ± 0.13
BC12	1.14 ± 0.08	0.11 ± 0.01	1.56 ± 0.02
BC13	5.45 ± 0.09	0.14 ± 0.00	2.65 ± 0.12
BC14	2.77 ± 0.05	0.10 ± 0.01	1.37 ± 0.04

BC15	1.79 ± 0.10	0.10 ± 0.01	1.22 ± 0.02
BC16	2.85 ± 0.07	0.11 ± 0.00	1.66 ± 0.09
BC17	4.14 ± 0.08	0.10 ± 0.01	1.89 ± 0.12
BC18	4.40 ± 0.05	0.15 ± 0.00	3.77 ± 0.09
BC19	2.86 ± 0.03	0.10 ± 0.00	2.35 ± 0.17
BC20	3.14 ± 0.08	0.09 ± 0.00	1.96 ± 0.11
BC21	2.56 ± 0.07	0.06 ± 0.01	0.81 ± 0.02
BC22	5.82 ± 0.03	0.18 ± 0.01	5.56 ± 0.04
BC23	3.25 ± 0.08	0.07 ± 0.01	1.35 ± 0.02
BC24	4.71 ± 0.07	0.10 ± 0.01	2.15 ± 0.08
BC25	4.62 ± 0.03	0.09 ± 0.01	2.26 ± 0.02
BC26	3.26 ± 0.07	0.09 ± 0.00	1.11 ± 0.01
BC27	1.41 ± 0.05	0.08 ± 0.00	0.89 ± 0.07
BC28	1.14 ± 0.05	0.08 ± 0.00	0.93 ± 0.03
BC29	1.84 ± 0.05	0.08 ± 0.00	1.19 ± 0.02
BC30	3.85 ± 0.05	0.09 ± 0.00	1.43 ± 0.04
BC31	4.82 ± 0.07	0.11 ± 0.01	1.82 ± 0.04
BC32	4.85 ± 0.05	0.11 ± 0.01	1.92 ± 0.04
BC33	4.71 ± 0.07	0.11 ± 0.00	1.59 ± 0.01
BC34	5.13 ± 0.07	0.10 ± 0.00	2.70 ± 0.05
BC35	4.60 ± 0.05	0.10 ± 0.01	1.85 ± 0.03
BC36	4.49 ± 0.03	0.15 ± 0.01	1.45 ± 0.02
FUN	5.12 ± 0.07	0.15 ± 0.01	2.83 ± 0.02
PAT	4.55 ± 0.05	0.13 ± 0.01	1.76 ± 0.04
POL	5.19 ± 0.03	0.16 ± 0.01	1.92 ± 0.04
CHIP	4.73 ± 0.07	0.12 ± 0.02	2.69 ± 0.02
STC	2.48 ± 0.08	0.09 ± 0.00	1.20 ± 0.02
NOB	3.28 ± 0.05	0.10 ± 0.00	0.64 ± 0.02
HB1	1.95 ± 0.09	0.16 ± 0.01	1.04 ± 0.03
HB2	2.75 ± 0.08	0.09 ± 0.00	1.57 ± 0.02
HB3	4.07 ± 0.10	0.07 ± 0.01	1.71 ± 0.02
HB4	3.68 ± 0.05	0.11 ± 0.00	1.40 ± 0.02
HB5	2.94 ± 0.17	0.10 ± 0.01	1.42 ± 0.02
HB6	0.29 ± 0.10	0.13 ± 0.01	1.22 ± 0.02
HB7	2.41 ± 0.10	0.09 ± 0.00	1.23 ± 0.02
HB8	2.00 ± 0.33	0.14 ± 0.01	1.48 ± 0.06
HB9	2.74 ± 0.08	0.10 ± 0.00	1.42 ± 0.02
HB10	0.79 ± 0.10	0.10 ± 0.00	1.32 ± 0.02
HB11	4.48 ± 0.04	0.16 ± 0.01	1.49 ± 0.02
HB12	4.82 ± 0.09	0.09 ± 0.01	1.95 ± 0.03
HB13	3.13 ± 0.10	0.10 ± 0.02	1.27 ± 0.01
HB14	5.11 ± 0.05	0.12 ± 0.00	1.67 ± 0.02
HB15	4.47 ± 0.07	0.09 ± 0.00	1.40 ± 0.02
HB16	2.52 ± 0.07	0.09 ± 0.00	0.91 ± 0.03
HB17	4.79 ± 0.07	0.10 ± 0.00	1.30 ± 0.04
HB18	4.09 ± 0.03	0.10 ± 0.00	1.45 ± 0.02
HB19	2.74 ± 0.05	0.08 ± 0.00	0.87 ± 0.04
HB20	4.69 ± 0.09	0.10 ± 0.00	1.37 ± 0.02
HB21	5.13 ± 0.05	0.10 ± 0.00	1.40 ± 0.02
HB22	4.62 ± 0.03	0.10 ± 0.00	1.27 ± 0.01
HB23	4.01 ± 0.04	0.10 ± 0.00	1.82 ± 0.02
HB24	3.25 ± 0.08	0.09 ± 0.00	1.19 ± 0.01
HB25	2.76 ± 0.10	0.09 ± 0.00	1.22 ± 0.01

HB26	3.66 ± 0.07	0.10 ± 0.00	1.52 ± 0.03
HB27	4.79 ± 0.09	0.09 ± 0.00	1.56 ± 0.02
HB28	4.72 ± 0.06	0.10 ± 0.00	1.74 ± 0.04

Values are means ± SD values of at least three replicates. There was no significant difference among individuals in TAA, TFC and TPC according to the Kruskal-Wallis test.

Table S3. Pearson correlation coefficients of antioxidant properties. The respective p-values are shown in brackets. The significance level alpha is 0.05.

Variables	TAA (mg GAE/g fl)	TFC (mg CE/g fl)	TPC (mg GAE/g fl)
**TAA (mg GAE/g fl)**	1 (0)	0.387 (0.001)	0.352 (0.003)
**TFC (mg CE/g fl)**	0.387 (0.001)	1 (0)	0.826 (<0.0001)
**TPC (mg GAE/g fl)**	0.352 (0.003)	0.826 (<0.0001)	1 (0)

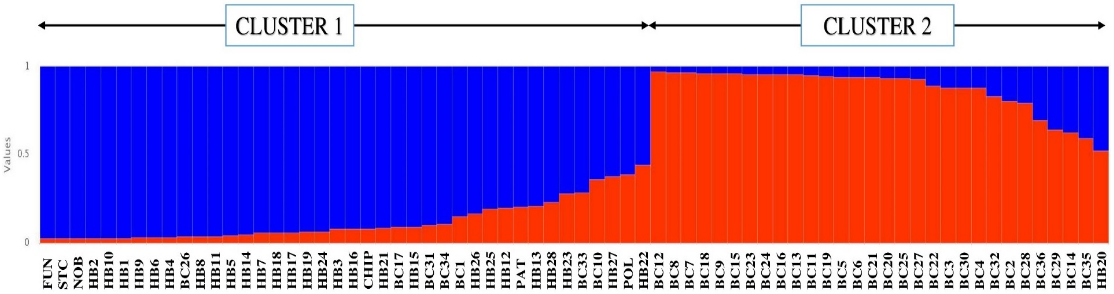

Figure S1. Distribution of blueberry genotypes as per STRUCTURE analysis (K = 2) based on EST-SSR primer pairs. The genotypes are represented as vertical bars, and the colour represents different clusters (see Tables 1 and 2 for genotype label).

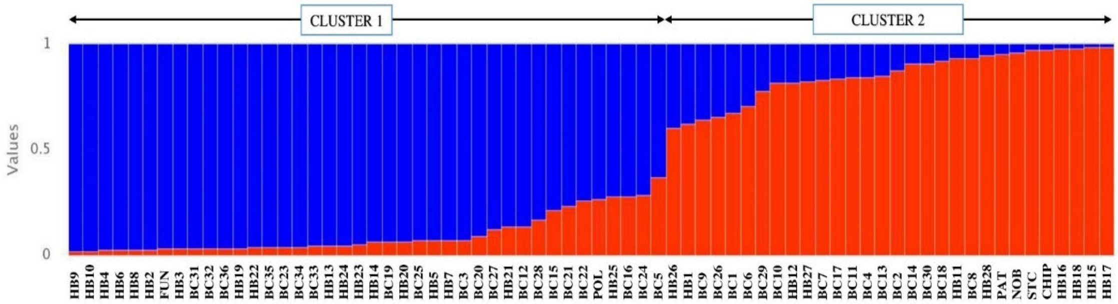

Figure S2. Distribution of blueberry genotypes as per STRUCTURE analysis (K = 2) based on genomic (G-) SSR primer pairs. The genotypes are represented as vertical bars, and the colour represents different clusters (see Tables 1 and 2 for genotype label).

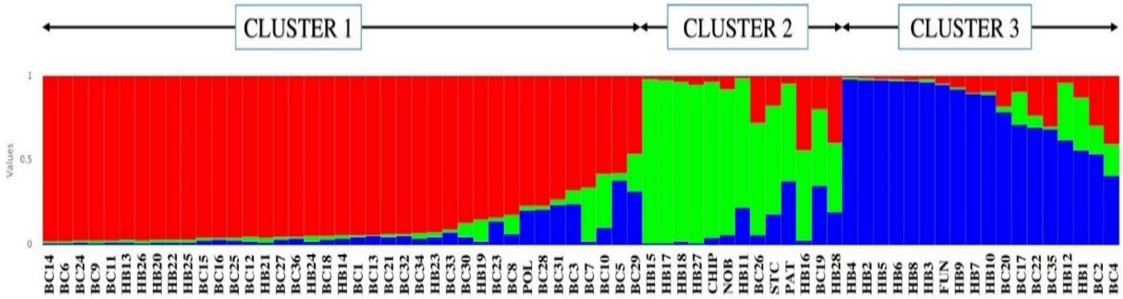

Figure S3. Distribution of blueberry genotypes as per STRUCTURE analysis (K = 3) based on EST-PCR primer pairs. The genotypes are represented as vertical bars, and the colour represents different clusters (see Tables 1 and 2 for genotype label).

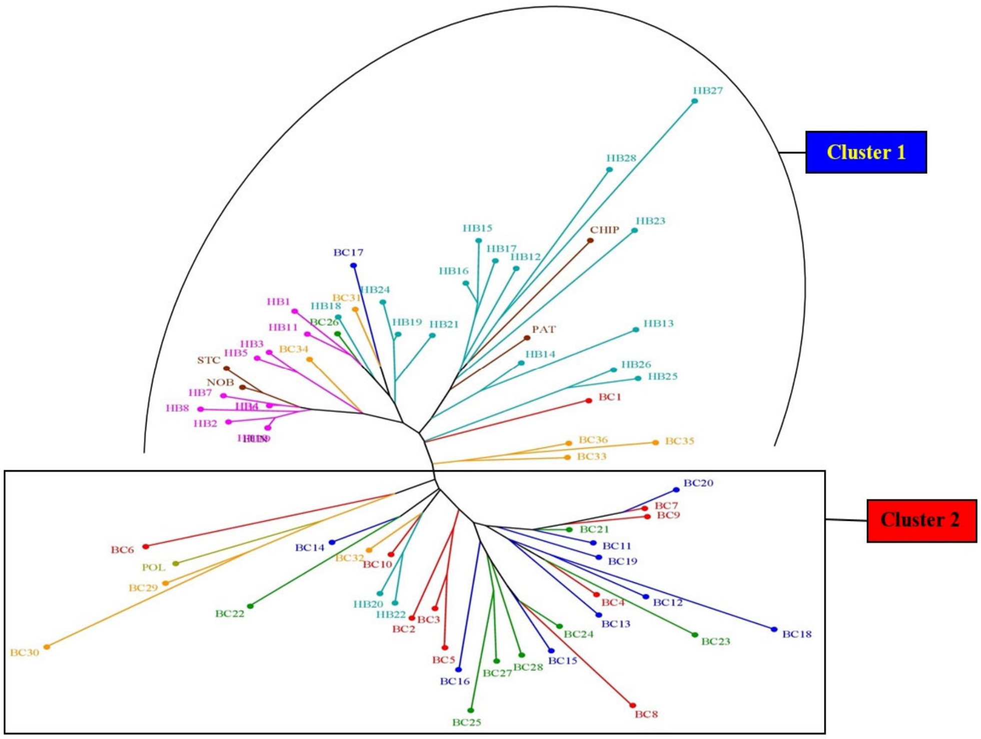

Figure S4. Dendrogram produced by using the unweighted neighbour-joining (NJ) method based on genetic dissimilarity produced by EST-SSR markers among blueberry genotypes. The colour of the branches indicates different groups (see Tables 1 and 2 for genotype label).

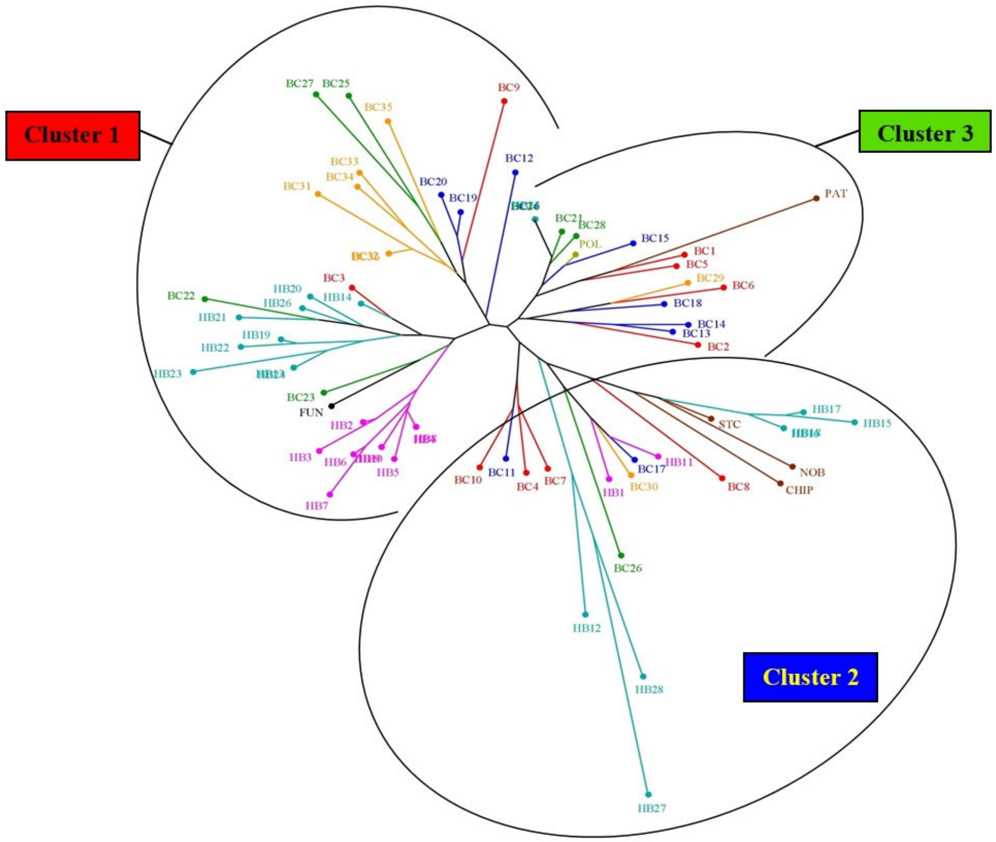

Figure S5. Dendrogram produced by using the unweighted neighbour-joining (NJ) method based on genetic dissimilarity produced by genomic (G-) SSR markers among blueberry genotypes. The colour of the branches indicates different groups (see Tables 1 and 2 for genotype label).

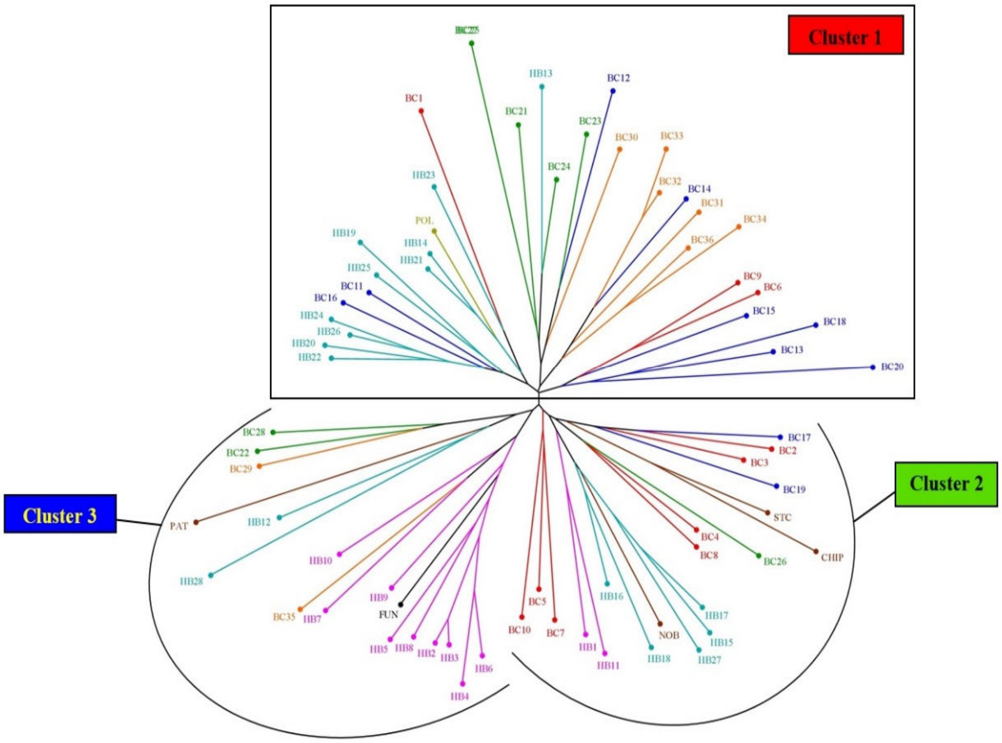

Figure S6. Dendrogram produced by using the unweighted neighbour-joining (NJ) method based on genetic dissimilarity produced by EST-PCR markers among blueberry genotypes. The colour of the branches indicates different groups (see Tables 1 and 2 for genotype label).

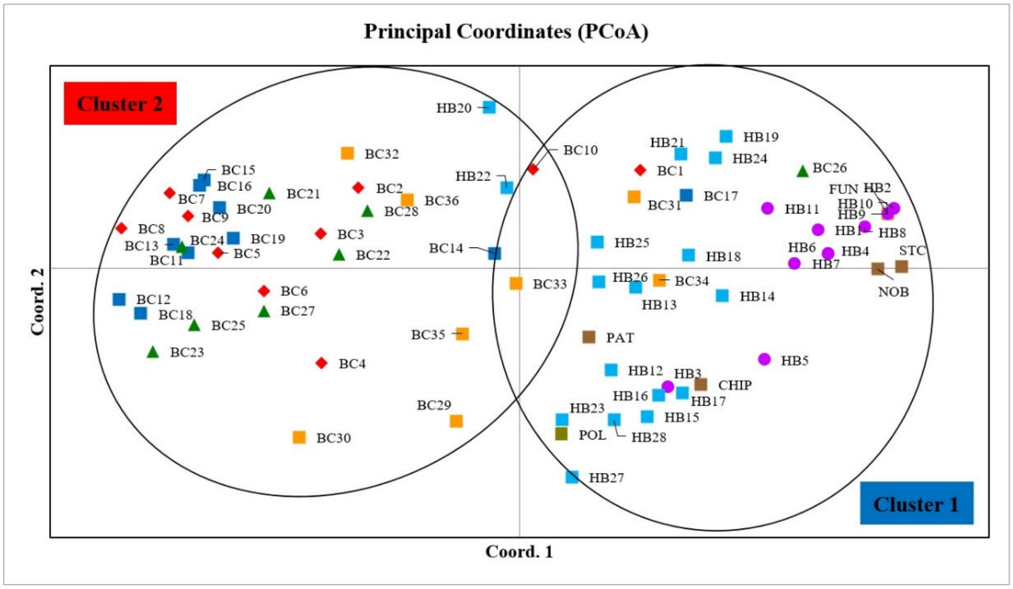

Figure S7. 2D principle coordinate analysis (PCoA) plot of blueberry genotypes using genetic distance matrix produced by EST-SSR markers. The colour and shape of the points indicate different groups (see Tables 1 and 2 for genotype label).

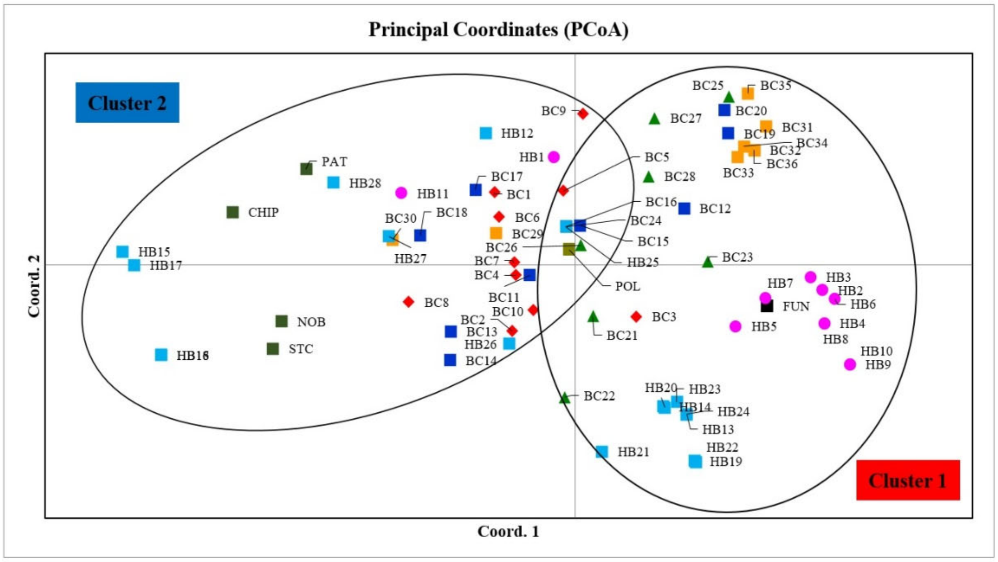

Figure S8. 2D principle coordinate analysis (PCoA) plot of blueberry genotypes using genetic distance matrix produced by genomic (G-) SSR markers. The colour and shape of the points indicate different groups (see Tables 1 and 2 for genotype label).

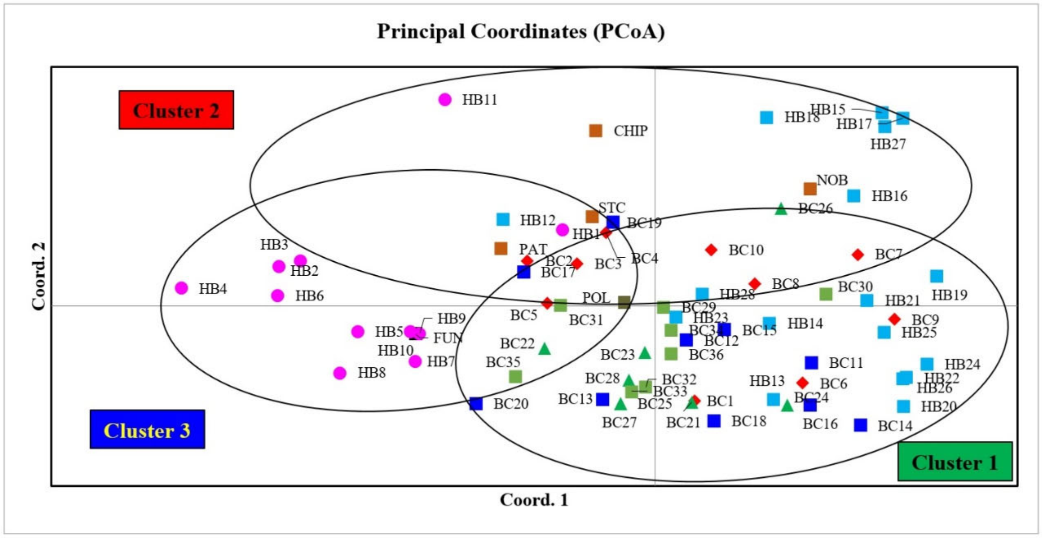

Figure S9. 2D principle coordinate analysis (PCoA) plot of blueberry genotypes using genetic distance matrix produced by EST-PCR markers. The colour and shape of the points indicate different groups (see Tables 1 and 2 for genotype label).
